# An Alternative Polymer Material to PVDF Binder and Carbon Additive in Li‐Ion Battery Positive Electrode

**DOI:** 10.1002/advs.202409403

**Published:** 2024-10-21

**Authors:** Ivone Marselina Nugraha, Jacob Olchowka, Cyril Brochon, Delphine Flahaut, Mélanie Bousquet, Benjamin Cabannes‐Boue, Rafael Bianchini Nuernberg, Éric Cloutet, Laurence Croguennec

**Affiliations:** ^1^ Univ. Bordeaux CNRS Bordeaux INP ICMCB, UMR 5026 Pessac F‐33600 France; ^2^ Univ. Bordeaux CNRS Bordeaux INP LCPO, UMR 5629 Pessac F‐33600 France; ^3^ RS2E Réseau Français sur le Stockage Electrochimique de l'Energie CNRS FR 3459 Amiens Cedex 1 80039 France; ^4^ ALISTORE‐ERI FR CNRS 3104 33 Rue Saint‐Leu Amiens Cedex 1 80039 France; ^5^ IPREM – UMR 5254 UPPA CNRS – ECP Technopole Helioparc 2, av. Pdt P. Angot PAU Cedex 09 64 053 France

**Keywords:** Li‐ion battery positive electrode, mixed conducting polymer, olivine LiFe_1‐y_Mn_y_PO_4_, Poly(3,4‐ethylenedioxythiophene) (PEDOT), polymer binder

## Abstract

Li‐ion battery performance relies fundamentally on modulation at the microstructure and interface levels of the composite electrodes. Correspondingly, the binder is a crucial component for mechanical integrity of the electrode, serving to interconnect the active material and conductive additive and to firmly attach this composite to the current collector. However, the commonly used poly(vinylidenefluoride) (PVDF) binder presents several limitations, including the use of toxic solvent during processing, a low electrical conductivity which for compensation requires the addition of carbon black, and weak interactions with active materials and collectors. This study investigates Poly(3,4‐ethylenedioxythiophene):poly[(4‐styrenesulfonyl) (trifluoromethylsulfonyl) imide] (PEDOT:PSSTFSI) as an alternative binder and conductive additive, in replacement of both PVDF and carbon black, in Li‐ion batteries with LiFe_0.4_Mn_0.6_PO_4_ at the positive electrode. Complex PEDOT:PSSTFSI significantly improves the electronic conductivity and lithium diffusion coefficient within the electrode, in comparison to standard PVDF binder and carbon black. This enhances significantly the electrochemical performance at high C‐rates and for high active mass loading electrodes. Furthermore, an excellent long‐range cyclability is achieved.

## Graphical abstract

Binders in Li‐ion battery play an important role to ensure mechanical integrity and interface modulation of electrodes. This study explores PEDOT:PSSTFSI as an alternative mixed conductive binder in positive electrodes. The use of this polymer enhances conductivity and lithium diffusion, facilitates faster charge/discharge rates at high current densities, and promotes stability over extended cycles. These results introduce a new binder to substitute both PVDF and carbon black in conventional formulations.

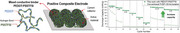

## 1 Introduction

Li‐ion battery has emerged these last decades as a ground‐breaking innovation in the energy storage field, enabling greater mobility and productivity in our daily lives. As the demand for high‐performance batteries with increased power and energy density continues to rise, extensive research is still currently done to explore high‐capacity and/or high‐voltage active materials, advanced electrolytes, or novel battery technologies such as all solid‐state battery.^[^ ^1^, ^2^, ^3^, ^4^, ^5^, ^6^ ^]^ Dealing with active materials, the introduction of LiFe_x_Mn_1‐x_PO_4_ with optimized morphology and carbon coating as a new positive electrode material in lithium‐ion batteries represents a significant milestone in the field of energy storage technology.^[^ ^7^, ^8^, ^9^, ^10^, ^11^ ^]^ Partial substitution of Fe by Mn in the olivine structure of LiFePO_4_, as first proposed in 1997 by Padhi et al., ^[^ ^12^, ^13^, ^14^, ^15^ ^]^ permits to increase the working potential from 3.45 to 4.10 V versus Li^+^/Li. Maintaining combination between i) this higher working voltage of LiMnPO_4_, ii) excellent rate performance well‐known for carbon‐coated LiFePO_4_, iii) good chemical and thermal stability of polyanionic frameworks and iv) a structure containing only sustainable transition metal elements, has made of LiFe_x_Mn_1‐x_PO_4_ a new highly attractive material. In good agreement with results reported by Yamada et al., manganese‐rich LiFe_0.4_Mn_0.6_PO_4_ appears as the best compromise among LiFe_x_Mn_1‐x_PO_4_ compositions, with a more robust stability upon cycling compared to LiMnPO_4_ and a ≈20% higher energy density than LiFePO_4_.^[^ ^11^ ^]^

However, although the optimization of active material is important, the overall performance of Li‐ion battery relies also fundamentally on modulation of microstructure and interfaces within the composite electrodes. This involves optimizing porosity for an efficient impregnation of the electrolyte, electronic percolation, and thus connectivity between the active material, the conductive additive, and the current collector, and processing in order to maintain adhesion and integrity of the electrode despite high mass loading.^[^ ^16^ ^]^ The role of an efficient binder is crucial as it ensures mechanical integrity within the composite electrodes, interconnection between the active material and the electronic conductive additive, and permits the adhesion of the active material to the current collector while being flexible enough to accommodate volume changes during cycling without fracturing.^[^ ^17^, ^18^ ^]^ To achieve optimal binding properties, it is necessary to adjust the type and strength of interactions by modifying the chemistry and functionalities of the binder considering the battery chemistry and thus the nature of the active materials. Indeed, the binder is one of the main components responsible for capacity loss and reduced coulombic efficiency of a Li‐ion battery, when poor interconnectivity leads to loss of electrical percolation within the electrode, but also to possible decohesion of the active material with ultimately delamination from the current collector.^[^ ^19^, ^20^ ^],^ Therefore, optimization of electrode composition and formulation for high‐quality homogeneous slurry, which determines the overall structure of the composite electrodes, is crucial for achieving the best electrochemical performance.^[^ ^21^, ^22^, ^23^ ^]^

Currently, one of the most commonly used binders at the positive electrode in standard Li‐ion batteries is Poly(vinylidenefluoride) (PVDF). However, PVDF exhibits several limitations, including its non‐polar structure, which restricts the range of compatible solvents. This leads to the reliance on N‐Methyl‐2‐pyrrolidone (NMP) during the casting process, creating challenges for recycling PVDF in end‐of‐life batteries and contributing to environmental concerns.^[^ ^24^ ^]^ Further it allows only the formation of weak intermolecular/physical adsorption interactions with active materials and current collectors, which can trigger loss of contact and therefore significant capacity loss at an elevated temperature (above 55 ^°^C). Its electrically insulating nature requires the addition of carbon additives to enhance the electrical conductivity of the composite electrode.^[^ ^18^, ^20^ ^]^

Although the use of PVDF or other binders requires the addition of an electronic conductive additive to the electroactive material, this approach comes with several drawbacks. Incorporating both a binder and a conductive additive increases the complexity and cost of the electrode manufacturing process, as it requires extra steps to achieve a homogeneous mixture. Additionally, the binder and conductive additive take up valuable space within the electrode that could otherwise be used for more electroactive material, ultimately reducing the battery's energy capacity. To overcome these insulating properties, reduce the need for conductive additives, and improve binder performance, the development of new binders that are both electronically and ionically conductive is a promising approach.

Nowadays, conducting polymers like polypyrrole, polyaniline, and polythiophene have been widely investigated for multiple purposes in energy storage applications ranging from conductive additives or binders to active materials or surface modification of active materials, etc.^[^ ^25^, ^26^ ^]^ Poly(3,4‐ethylenedioxythiophene)‐poly(styrenesulfonate) (PEDOT:PSS) is commercially available and has for instance been widely investigated as conductive binder for a wide range of electrode active materials including LiFePO_4_, LiCoO_2_, Li_4_Ti_5_O_12_, LiNi_1/3_Mn_1/3_Co_1/3_O_2_, LiNi_0.5_Mn_1.5_O_4_, graphite, and silicon anodes.^[^ ^18^, ^27^ ^]^ Nevertheless, despite promising results, its use requires the addition of conventional non‐conductive binders such as carboxymethyl cellulose (CMC), PVDF or styrene butadiene rubber (SBR) along with carbon additives, to compensate for still too much limited electrical conductivity and mechanical properties, lowering thus the gravimetric energy density of the final electrode.^[^ ^25^, ^28^, ^29^, ^30^, ^31^ ^]^

Conducting polymer PEDOT itself can be doped or complexed with other polyanions/polyelectrolytes to optimize its performance as binder in the electrode composite. One of the most interesting polyelectrolytes for this purpose would be the poly[(4‐styrenesulfonyl) (trifluoromethylsulfonyl) imide] (PSSTFSI).^[^ ^31^ ^]^ The anionic (trifluoromethylsulfonyl) imide (TFSI) groups carried by the styrene chain, unlike the sulfonates in PEDOT:PSS, are substantially dissociated because of extended delocalization greatly increasing the ionic conductivity of about one order of magnitude as compared to PSS.^[^ ^31^ ^]^ Moreover, Armand and Bonnet,^[^ ^32^ ^]^ pioneers in the synthesis of polystyrene‐bearing TFSI anion (PSSTFSI polyanion), have applied PSSTFSI polyanion as a single‐ion polymer electrolyte in solid state battery, highlighting its ability to conduct Li^+^ cation. The use of complex PEDOT:PSSTFSI has been investigated in optoelectronic devices applications,^[^ ^33^, ^34^ ^]^ with promising features demonstrated in terms of mixed conductivity, thermal stability, and mechanical properties, but to the best of our knowledge it has never been tested in batteries.

Herein, we propose a novel mixed conductive PEDOT:PSSTFSI to replace both PVDF binder and carbon electronic additives in the electrode formulation used for LiFe_0.4_Mn_0.6_PO_4_. PEDOT:PSSTFSI synthesized by oxidative polymerization in dispersion in water, has both suitable binding properties to ensure mechanical stability of the electrode and appropriate electrical and ionic conductivity making it possible to dispense with the addition of carbon traditionally used for electronic percolation within the electrode.

## 2 Results and Discussion

### 2.1 Preparation of Alternative Mixed Conductive Polymer Binder

The preparation of the mixed conductive PEDOT:PSSTFSI polymer began with the synthesis of a styrene monomer with a TFSI functional group, which provides ionic conduction. The obtained monomer, potassium (4‐styrenesulfonyl)(trifluoromethylsulfonyl)imide (SSTFSIK), is shown in Figure S1 (Supporting Information) with also more details on its structural characterization by ^1^H‐NMR, ^13^C‐NMR, and ^19^F‐NMR in Figure S2 (Supporting Information).^[^ ^32^, ^35^ ^]^ The light‐yellow synthesized solid SSTFSIK was then polymerized in dimethylformamide (DMF) by controlled radical polymerization following the reversible addition‐fragmentation chain‐transfer (RAFT) mechanism,^[^ ^32^, ^36^ ^]^ with a target molecular weight (Mw) of 100 kDa that was analyzed by Size Exclusion Chromatography (SEC) in DMF (+ LiBr 1g L^−1^) as the eluent. The synthesized PSSTFSIK was obtained with a Mw of 134 000 g mol^−1^ and a dispersity (Ð) of 2.1 (Figure S3 and Table S1, Supporting Information). Thermogravimetric analyses (TGA) and differential scanning calorimetry (DSC) were also done for PSSTFSIK sample, the results are shown in Figures S4 and S5 (Supporting Information), respectively.

**Scheme 1:**
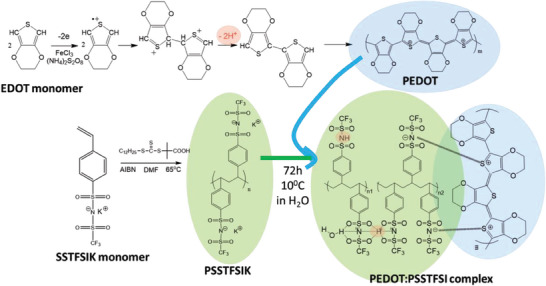
Schematic description of PEDOT:PSSTFSI complex preparation.

The PEDOT:PSSTFSI complex was synthesized by a classical oxidative polymerization of 3,4‐ethylenedioxythiophene (EDOT) in the aqueous PSSTFSIK solution in deionized water as represented in **Scheme** **1**.^[^ ^33^, ^34^ ^]^ The molar ratio of EDOT:SSTFSI was 0.5, while the molar ratio of the oxidants (NH_4_)_2_S_2_O_8_:FeCl_3_ was 3.5 and that of (NH_4_)_2_S_2_O_8_ to EDOT was 2.3. After 72 h at 10 °C, the PEDOT:PSSTFSI dispersion was purified using anionic and cationic ion exchange resins, and then concentrated using an ultrafiltration cell until maximum water could be removed and dark blue gel obtained. Finally, the water contained in PEDOT:PSSTFSI was removed by freeze‐drying, as residual water can react not only with the positive electrode material but also with the electrolyte. A dark blue flaky powder was obtained and analyzed by TGA, under nitrogen flow to determine its water content that was ≈1.4 wt.% (Figure S6, Supporting Information). The low residual water content may be attributed to hydrogen bonding in the complexation of PEDOT:PSSTFSI, as supported by the XPS results discussed in the following paragraph. Meanwhile, the DSC results (Figure S7, Supporting Information) showed a glass transition temperature ≈53 °C at a heating rate of 10 °C min^−1^, with no melting peak observed, indicating that PEDOT:PSSTFSI is predominantly amorphous.

**Figure 1:**
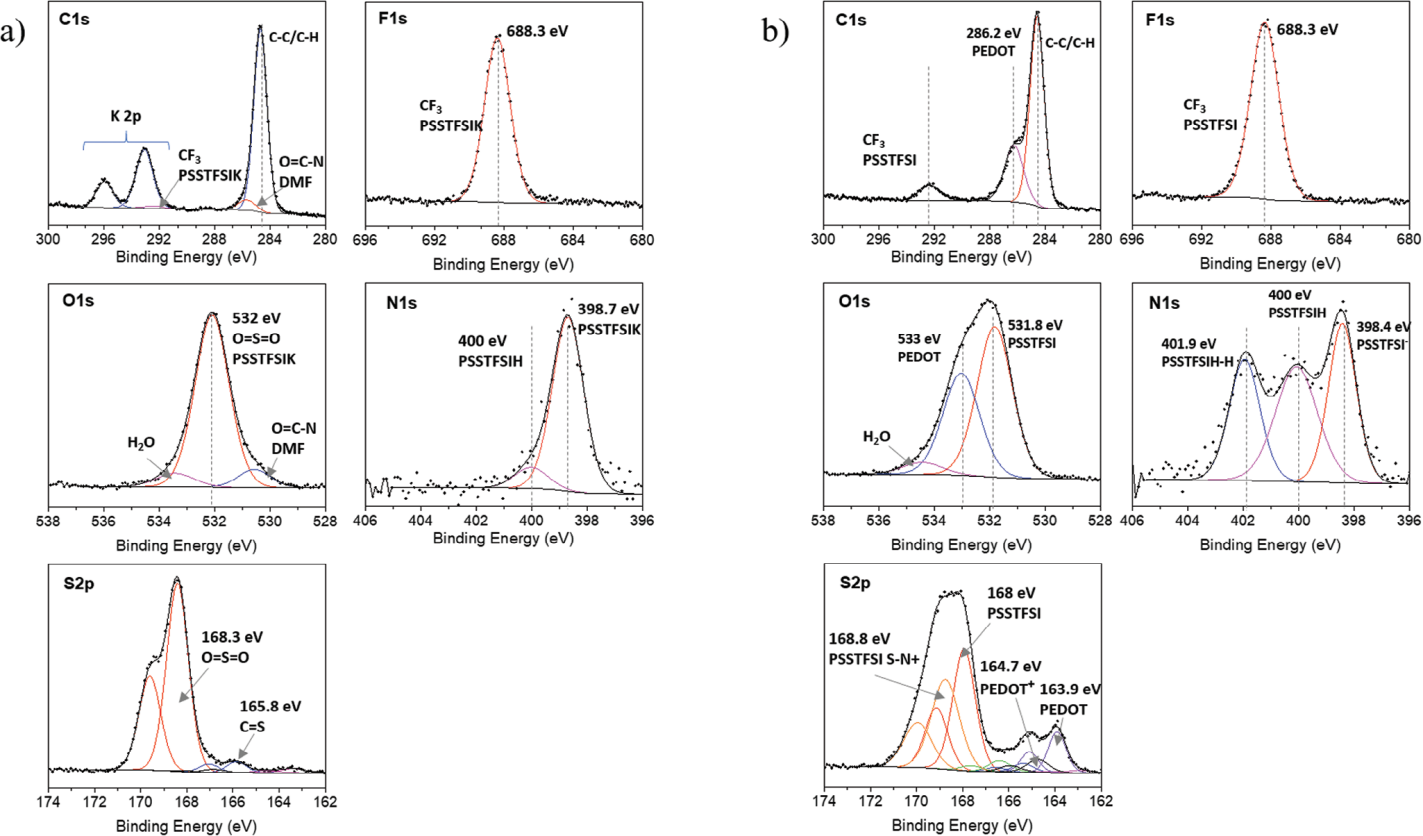
C1s, F1s, O1s, N1s, and S2p XPS spectra and their desummation for a) PSSTFSIK and b) PEDOT:PSSTFSI.

To understand this complexation and the binding properties of PEDOT:PSSTFSI, X‐ray photoelectron spectroscopy (XPS) measurements were carried out on PSSTFSIK and complex PEDOT:PSSTFSI (see Figures S8 and S9, Supporting Information for XPS survey spectra). As shown in **Figure** **1**, a detailed analysis of the C1s, F1s, O1s, N1s and S2p XPS spectra was performed.

The core peaks of the PSSTFSIK will be described at first. The C1s spectrum reveals the CF_3_ groups at 292.3 eV, which are overlapped with K2p doublet, a minor contribution of the oxygenated species issued from the contamination at 285.6 eV and a large one associated to both adventitious carbon and carbon of the polymer chain. The signature of the PSSTFSIK is well characterized by a single component in the F1s and O1s core peak located at 688.3 and 532 eV, respectively. Traces of H_2_O and DMF solvents are observed in O1s and C1s core peak, their quantity is very low (see Table S2 (Supporting Information) for the quantification in supporting information). The N1s spectrum of PSSTFSIK is decomposed in two components at 398.7 eV, corresponding to mainly negatively charged nitrogen in PSSTFSI^−^ that interacts with K^+^, and a less intense one at 400 eV attributed to nitrogen interacting with remaining H^+^ and forming PSSTFSIH block. Due to the spin‐orbit coupling for the p orbitals, the S2p spectrum is decomposed in several 2p_3/2‐1/2_ doublets for identified chemical environments. The sulfonyl groups of PSSTFSIK chain are associated to the doublet at 168.3–169.5 eV. A second minor doublet observed at 165.8–167.0 eV might correspond to the end chain of PSSTFSIK that consists of trithiocarbonate functional groups from the RAFT agent (2‐(Dodecylthiocarbonothioylthio)‐2‐methyl propionic acid).

The formation of the PEDOT:PSSTFSI complex is validated by the assignment of new components observed in the core peaks spectra to the environment present in the PEDOT polymer. Indeed, the peak at 286.2 eV of C1s core peak is a signature of C─O cyclic bond of PEDOT. The F1s spectrum shows no change in peak signature and binding energy, indicating the similar nature of CF_3_ binding in PSSTFSIK and PEDOT:PSSTFSI. Moreover, the potassium has been removed during the preparation of the complex PEDOT:PSSTFSI as confirmed by the vanishing of K2p signals on C1s spectrum. The addition of a second component at 533 eV peak in the O1s core spectrum of PEDOT:PSSTFSI is characteristic of oxygen in the cyclic C─O bond of PEDOT. A significant change is observed in the N1s spectrum of PEDOT:PSSTFSI. Three components are detected not due to PEDOT chain (nitrogen free) but to the binding properties of the PEDOT:PSSTFSI complex itself and also to the presence of bound water forming hydrogen bonds between PSSTFSI backbone. The peak at 400 eV possesses the same assignment that in the PSSTFSIK and is still attributed to nitrogen interacting with remaining H^+^ and forming PSSTFSIH block. The extra peak at 401.9 eV corresponds to the interaction of PSSTFSIH moieties with other PSSTFSIH chains in the complex PEDOT:PSSTFSI. The 398.4 eV peak is related to negatively charged nitrogen in PSSTFSI^−^ interacting with positively charge PEDOT^[^ ^37^ ^]^ that is consistent with the slight shift toward lower binding energies comparing with the nitrogen in the PSSTFSIK (See Figure S10, Supporting Information). The ratio of (SSTFSI^−^/SSTFSIH)_N1s_ is 0.9. The decomposition of the S2p core peak after the complex formation is based on the use of the experimental envelope, resulting from the desummation of the S2p spectrum of the PSSTFSIK to decompose the rest assigned to the contribution of the PEDOT chain and PEDOT/PSSTFSI interactions. The S2p signal characteristic of the PEDOT is composed of two doublets at 163.9–165.1 eV corresponding to neutral charged S in PEDOT and the second at 164.7–165.9 eV to positively charged S in PEDOT (see Figure S10, Supporting Information). An addition of a doublet at 168.8 eV for PSSTFSI chain in PEDOT:PSSTFSI is observed and corresponds to PSSTFSI forming hydrogen bond with water.

As a conclusion, the quantification of the different signals on the S2p core peaks leads to a ratio of EDOT:SSTFSI of 0.4 (see Table S3, Supporting Information), similar to EDOT:SSTFSI ratio that has been employed during synthesis. XPS indicates that the interaction between PEDOT and PSSTFSI is present in the form of a cation‐anion interaction from PSSTFSI^−^ with EDOT^+^ moieties identified by N1s and S2p core peaks. Moreover, after complexation, the PSSTFSI structure is preserved as the binding energy remains similar for PSSTFSIK and PEDOT:PSSTFSI.

**Figure 2:**
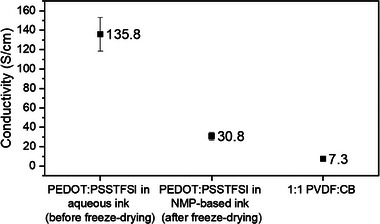
Electrical conductivity of PVDF:CB composite (1:1 weight ratio), and of PEDOT:PSSTFSI before and after freeze‐drying.

Before testing the potential of the PEDOT:PSSTFSI polymer as binder in the electrode formulation, its electrical conductivity was measured by the 4‐probe method on a bare casted polymer film, and compared to the 1:1 mass ratio of standard PVDF:carbon black (CB). As shown in **Figure** **2** and Table S4 (Supporting Information), the decrease in electrical conductivity of PEDOT:PSSTFSI after freeze‐drying is attributed to changes in the arrangement of its chains during the process. Freeze‐drying may disrupt the PEDOT chains or separate the conductive domains reducing the pathways for electron transport and thereby lowering the material's conductivity.

Nevertheless, the results clearly demonstrate that the electrical conductivity of complex PEDOT:PSSTFSI polymer after being freeze‐dried is 30.8 ± 3.93 S cm^−1^, i.e., still 4.2 times higher than that of the conventional 1:1 PVDF:CB composite that is only 7.3 ± 0.73 S cm^−1^. The high electrical conductivity of PEDOT:PSSTFSI is primarily due to the presence of PEDOT, a conjugated polymer that facilitates electron transport through its π‐conjugated system of aromatic thiophene rings. This significant enhancement of electrical conductivity makes PEDOT:PSSTFSI a highly promising candidate for replacing both the traditional PVDF binder and carbon black conductive additive in Li‐ion batteries application.

Considering that electrical conductivity is mostly dominate by the electronic conductivity due to PEDOT entities, the ionic conductivity of PEDOT:PSSTFSI was estimated applying a methodology based on electrochemical impedance spectroscopy (EIS). To this end, a PEDOT:PSSTFSI film was drop casted on a 9 mm^2^ masked area over a conductive fluorine‐doped tin oxide (FTO) electrode.^[^ ^38^, ^39^, ^40^ ^]^ As shown in Figure S11 (Supporting Information), an estimated ionic conductivity of 3.4 10^−5^ S.cm^−1^ at room temperature is obtained for PEDOT:PSSTFSI, surely several order of magnitude higher than that of the non‐conductive PVDF.^[^ ^41^ ^]^

A comparison of the nanomechanical properties of both polymers was also conducted using atomic force microscopy (AFM) on glass casted films, resulting in a Young's modulus of ±1.4 GPa for PVDF binder and ±5.8 GPa for PEDOT:PSSTFSI and highlighting a superiority of mechanical properties for this latter (Figure S12, Supporting Information).^[^ ^42^, ^43^ ^]^ This increase in Young's modulus could enhance the mechanical stability of the electrode during cycling, potentially reducing electrode degradation upon cycling. Additionally, the adhesion force for PEDOT:PSSTFSI (±18.1 nN), which is up to 2.5 times higher compared to PVDF (±7.8 nN) (Figure S13, Supporting Information), is a promising indicator of its suitability as a binder in battery applications. Furthermore, thanks to its polarity, the swellability of PEDOT:PSSTFSI is higher than for the PVDF binder (Figure S14, Supporting Information) while the highest mass intake of 1:1 PVDF:CB mixture originates from carbon black's high porosity. This higher swellability could improve ion transport, as it allows more electrolyte to be absorbed, which would be beneficial in the electrochemical performances of the battery.

The electrochemical stability of PEDOT:PSSTFSI was evaluated through cyclic voltammetry at various scan rates in potential range from 0–6 V versus Li^+^/Li. As shown in Figure S15 (Supporting Information), a lithium redox peak is observed for both samples in the 0–0.5 V range. A more intriguing finding emerges when focusing on the potential window of 2.5–6 V versus Li^+^/Li. Here, a higher current peak is seen for the sample with only electrolyte, which corresponds to electrolyte oxidation or degradation start from 4 V.^[^ ^44^ ^]^ Interestingly, in the case of PEDOT:PSSTFSI, this oxidation or degradation is less pronounced, as indicated by a lower current peak. Moreover, no additional oxidation peaks are detected for PEDOT:PSSTFSI, suggesting that it remains stable above 5 V and is not overoxidized within the 0–6 V versus Li^+^/Li range.

### 2.2 Rate Capability Performance

To validate the promising properties of PEDOT:PSSTFSI as binder, this latter was tested in two types of electrode formulation, the first one with classical weight ratio of active mass used at lab‐scale (85 wt.% of LiFe_0.4_Mn_0.6_PO_4_ (LFMP46) and 15 wt.% of PEDOT:PSSTFSI, named as “lab scale” hereafter) and the second one rich in active material (94 wt.% of LiFe_0.4_Mn_0.6_PO_4_ and 6 wt.% of PEDOT:PSSTFSI, named as “active material‐rich” hereafter) to be more in line with practical applications. Next, the energy storage performance of these electrodes were compared to those using conventional formulations in where the PEDOT:PSSTFSI weight ratio is replaced by a blend composed of an equivalent mass ratio of PVDF and carbon black. Prior to electrochemical testing, XRD analysis of the electrode was conducted to determine if there was any reactivity between LFMP46 and PEDOT:PSSTFSI during the composite electrode preparation. As shown in Figure S16 (Supporting Information), all the peaks observed in the pattern collected for LFMP46‐PEDOT:PSSTFSI correspond to those of the LFMP46 pristine powder, indicating no reactivity between LFMP46 and PEDOT:PSSTFSI. Furthermore, in accordance with DSC analysis of PEDOT:PSSTFSI (Figure S7, Supporting Information), the XRD analysis also indicates that PEDOT:PSSTFSI is predominantly amorphous, as no additional peaks at 2θ values of 12.4° and 25.9° – that are characteristic of crystalline PEDOT:PSS as reported by Yousefian et al.^[^ ^45^ ^]^ – were detected. SEM images of the composite electrodes were collected at the pristine state and after cycling, to compare their homogeneity with respect to the distribution of LFMP46 / PEDOT:PSSTFSI and LFMP46/PVDF/CB, and as shown in Figures S17 and S18 (Supporting Information), they appear very similar.

**Figure 3:**
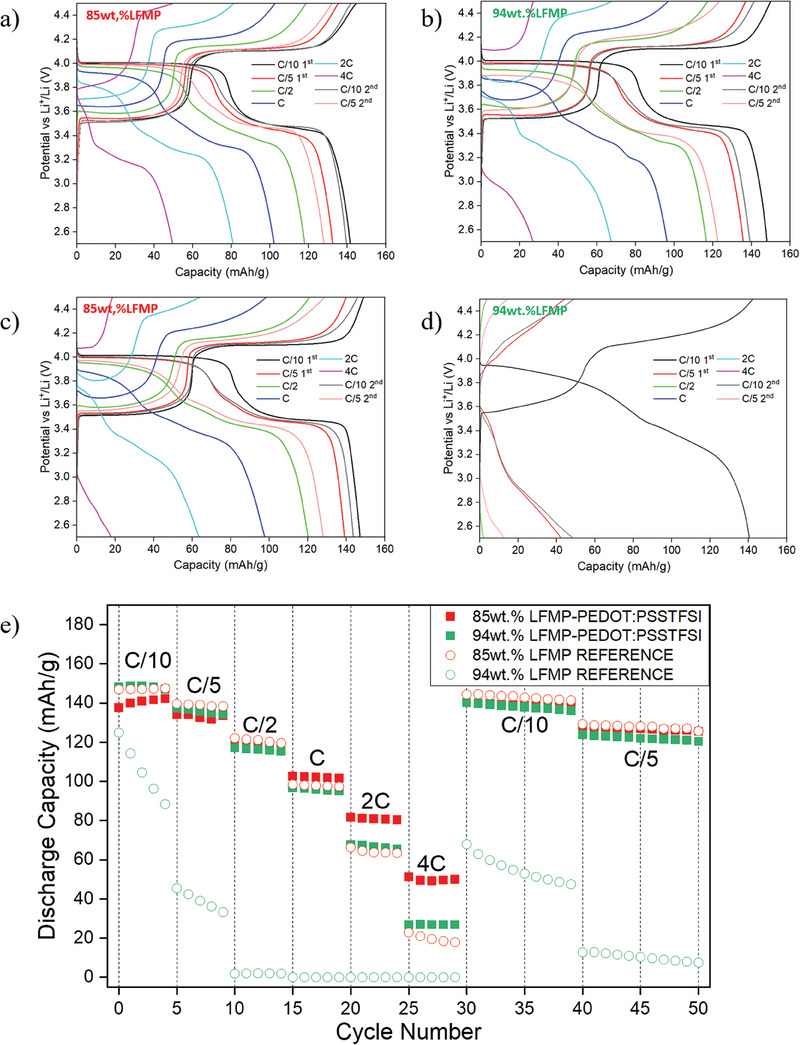
Galvanostatic charge/discharge curves obtained for a) 85 wt.% LFMP46 – 15 wt.% PEDOT:PSSTFSI, b) 94 wt.% LFMP46 – 6 wt.% PEDOT:PSSTFSI, c) 85 wt.% LFMP46 – 15 wt.% PVDF:CB (1:1) reference and d) 94 wt.% LFMP46 – 6 wt.% PVDF:CB (1:1) reference and e) corresponding discharge capacity evolution for the different LFMP46 electrode formulations. The assembled cells were cycled in Galvanostatic Cycling with Potential Limitation (GCPL) mode, with 5 cycles performed at each C rate (from C/10 to 4C) between 2.5 and 4.5 V versus Li^+^/Li.

**Figure** **3**a shows that the galvanostatic charge‐discharge curves obtained for the composite electrode LFMP46‐PEDOT:PSSTFSI at a C/10 rate clearly exhibit the typical electrochemical signature of LiFe_0.4_Mn_0.6_PO_4_ with the presence of two reversible plateaus at 3.4 and 4.1 V versus Li^+^/Li that are characteristic of Fe^2+^/Fe^3+^ and Mn^2+^/Mn^3+^ redox reactions, respectively. For both lab‐scale and active material‐rich formulations a specific capacity >140 mAh g^−1^ can be obtained at C/10, which is similar to that obtained with a PVDF:CB (1:1 weight ratio) formulation. More interestingly, composite electrodes containing the PEDOT:PSSTFSI binder exhibit greater capacity at high rates, compared with those based on PVDF:CB (1:1) formulation, highlighting the beneficial effect of good electronic conductivity of PEDOT:PSSTFSI polymer.

For instance, the composite electrode 85 wt.% LFMP46 – 15 wt.% PEDOT: PSSTFSI could still deliver a capacity of 50 mAh g^−1^ at 4C while only 20 mAh g^−1^ could be obtained for the corresponding 85 wt.% LFMP46 – 15 wt.% PVDF:CB (1:1) reference electrode. Furthermore, the initial capacity at C/10 is fully recovered after rate capability test, proving good cycling stability and absence of degradation upon high current densities. As shown in Figures S19 and S20 (Supporting Information), similar trends and performance are obtained for other studied electrode compositions with PEDOT: PSSTFSI.

The performance of the composite electrodes LFMP46‐PEDOT:PSSTFSI, with a higher mass percentage of active material of 94 wt.% LFMP46, is of particular interest (Figure 2e). These electrodes maintain high cyclability at high current densities up to 4C, whereas the corresponding reference electrode using PVDF:CB exhibit poor electrochemical performance already at C/5. These results demonstrate a significant improvement in capacity for active material‐rich electrode composition when using PEDOT:PSSTFSI as a mixed ionic and electronic conductor in replacement of both the PVDF binder and the carbon black conductive additive, allowing thus an increase of the battery's energy and power densities.

### 2.3 Diffusivity Properties

**Figure 4:**
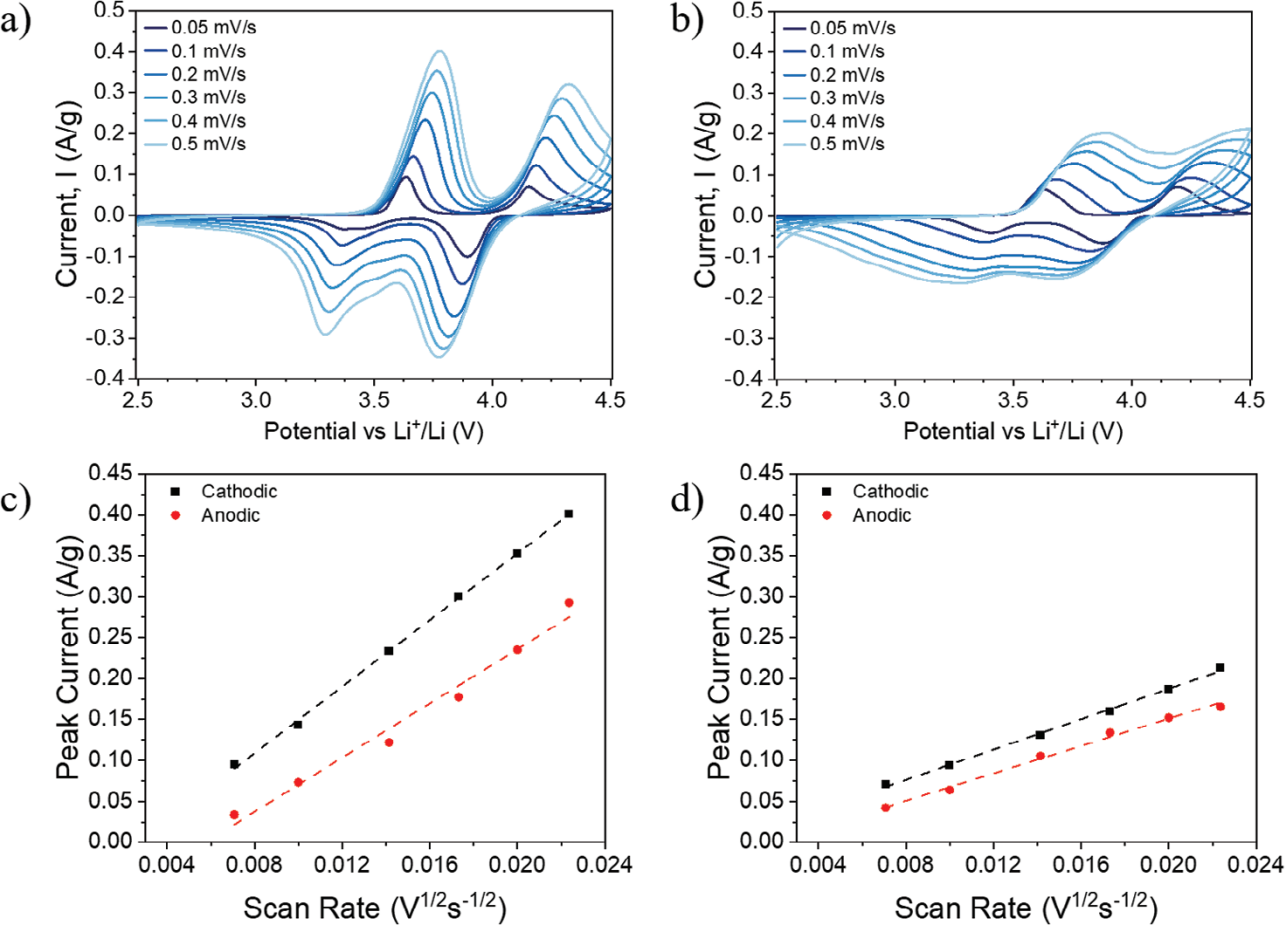
Cyclic voltammograms at various scan rates for a) 85 wt.% LFMP46 – 15 wt.% PEDOT:PSSTFSI and for b) 85 wt.% LFMP46 – 15 wt.% PVDF:CB (1:1) (reference), and the corresponding peak currents as function of square root of the scan rate (c,d).

To better understand these differences in terms of rate capability performance and diffusion properties depending on the nature of the electrode, either with the complex PEDOT:PSSTFSI polymer or with the standard PVDF as binder and carbon black as conductive additive, cyclic voltammetry (CV) experiments were carried out at various scan rates (**Figure** **4**a,b), enabling lithium diffusion into the composite electrodes to be estimated based on the Randles–Sevcik equation (see Supporting information and Table S5, Supporting Information for details).^[^ ^46^, ^47^ ^]^

The calculations of the apparent lithium diffusion coefficient were performed by plotting the peak maximum current as a function of the square root of scan rate (Figure 4c,d), which represents the average kinetics of all Li^+^ diffusion processes in the system.

As can be seen from the cyclic voltammograms (Figure 4a,b), both composites show two anodic peaks and two cathodic peaks, corresponding to the redox reaction of Fe^2+^/Fe^3+^ at 3.2–3.6 V and Mn^2+^/Mn^3+^ at 3.7–4.2 V versus Li^+^/Li.^[^ ^11^ ^]^ The Li^+^ diffusion current is compared using the peak current corresponding to the Fe^2+^/Fe^3+^redox reaction, as the high polarization and the cut‐off voltage at 4.5 V versus Li^+^/Li do not allow to fully observe for reference electrode the oxidation peak corresponding to the Mn^2+^/Mn^3+^ electrochemical activity. The D_Li+_ value determined for the composite electrode LFMP46‐PEDOT:PSSTFSI is one order magnitude higher in both cathodic (discharge) and anodic (charge) reactions (D_Li+_ = 2.2 × 10^−15^ and 1.4 × 10^−15^ cm^2^ s^−1^, respectively) to that determined for the composite electrode made using the conventional PVDF:CB formulation (D_Li+_ = 4.5 × 10^−16^ and 3.7 × 10^−16^ cm^2^ s^−1^, respectively). Since the electrode porosity and thickness are similar (see Tables S6 and S7, Supporting Information), the only difference between the two composite positive electrodes being the formulation and more specifically the binder, the higher lithium diffusion in LFMP46‐PEDOT:PSSTFSI electrode essentially originates from the higher ionic conductivity of PEDOT:PSSTFSI provided by the TFSI entities.

### 2.4 Cycling Stability

**Figure 5:**
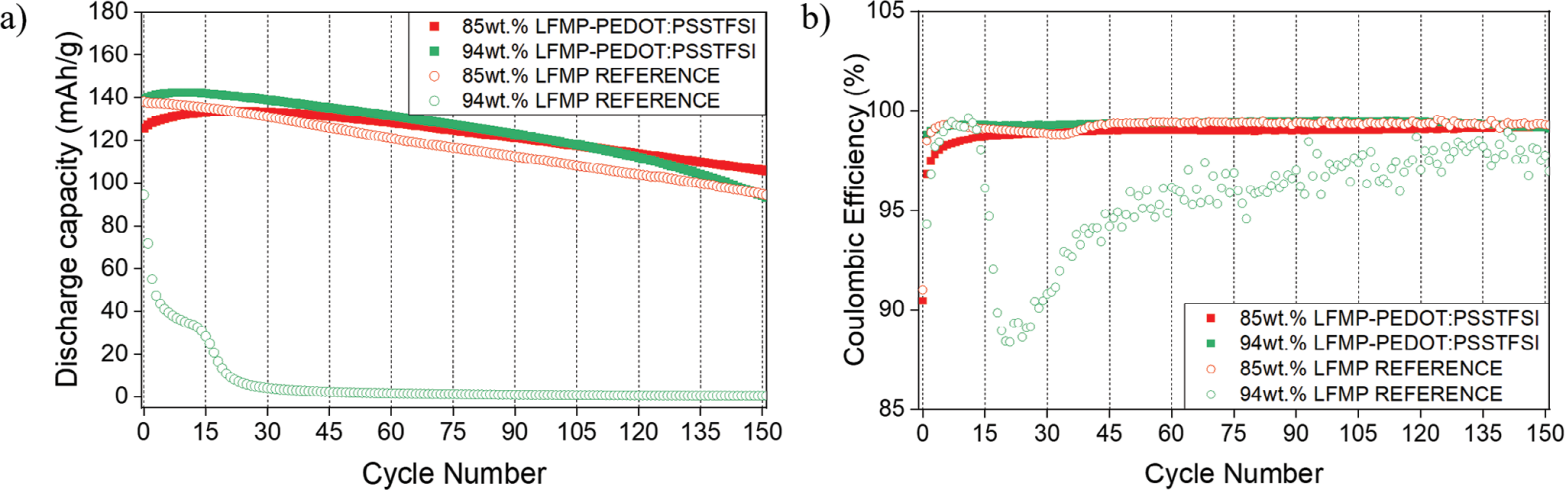
a) discharge capacity and b) coulombic efficiency over the long‐range cycling.

Beyond improving kinetics, the PEDOT:PSSTFSI binder provides more stable electrochemical performance over extended cycling compared to the PVDF binder. As shown in **Figure** **5**, for instance, after 150 cycles at C/5, the composite electrode 85 wt.% LFMP46 – 15 wt.% PEDOT:PSSTFSI still delivers 106 mAh/g whereas the classical electrode 85 wt.% LMFP46 – 15 wt.% PVDF:CB (1:1) delivers only 94 mAh g^−1^. This optimized capacity retention is even strongly accentuated for 94 wt.% active material loaded electrodes. For electrodes with classical formulation with PVDF:CB (1:1), the discharge capacity drops to almost 0 mAh g^−1^after 25 cycles, while for that made with PEDOT:PSSTFSI the discharge capacity remains at 110 mAh g^−1^ after 125 cycles. In addition, it can be observed that coulombic efficiency remains stable for the LFMP46‐PEDOT:PSSTFSI composite for the two percentages of LFMP46 active material studied. The slight increase in capacity observed during the first ten cycles is most probably due to an activation process associated to a gradual penetration of the electrolyte into the electrode porosity.

This hypothesis is supported by the evolution of polarization over cycles, which was evaluated by means of changes in dQ/dE for the two electrodes formulations with 85 wt.% active material (Figure S21, Supporting Information). Indeed, for LFMP46‐PEDOT:PSSTFSI electrode the overpotential for the low potential plateau progressively reduces upon the first cycles of the activation process before being stabilized with a polarization of ≈0.08 V whereas it continuously increases up to 0.2 V for the electrode with the classical PVDF formulation (Figures S21–S23, Supporting Information). Those best performance are maintained for LFMP46‐PEDOT:PSSTFSI at least over 150 cycles, although it is less obvious as the peak intensity decreases, in good agreement with a decrease in discharge capacity (Figures S21–S23, Supporting Information).

**Figure 6:**
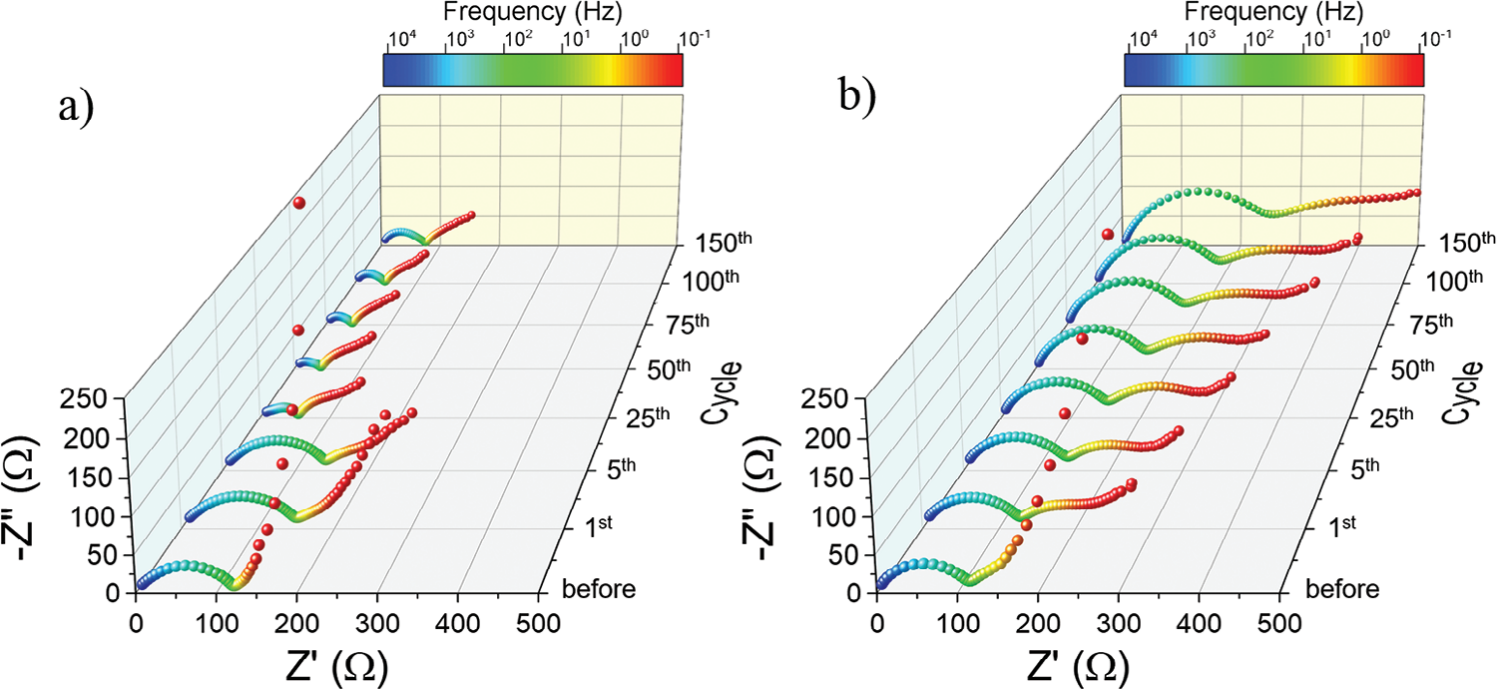
Impedance spectra evolution during prolonged cycling for a) 85 wt.% LFMP46 – 15 wt.% PEDOT:PSSTFSI and b) 85 wt.% LFMP46 – 15 wt.% PVDF:CB (1:1).

The evolution of the impedance spectra during cycling was performed to understand changes at the interfaces and the overall kinetics of the charge transfer and redox processes. As shown in **Figure** **6**, it is clear that the impedance spectra shape and magnitude are similar for both composite electrodes using PEDOT:PSSTFSI and PVDF:CB (1:1) for the first cycles, with a capacitive effect in low‐frequency range featured by a blocking phenomenon, which would be associated to the interface between the liquid electrolyte and the electrode. This capacitive phenomenon diminishes in the subsequent cycles and is replaced by a Warburg diffusion feature, as indicated by a 45^°^ line that corresponds to diffusion phenomena into the electrode. For the conventional PVDF:CB‐based composite electrode, the high frequencies semi‐circle which is often attributed to both interfacial characteristics of the solid electrolyte interface (SEI) and charge transfer resistance^[^ ^48^, ^49^, ^50^ ^]^ increases progressively upon cycling, whereas the PEDOT:PSSTFSI‐based composite electrode shows a significant decrease in total resistance upon cycling after the 5th cycle. As expected, these phenomena are in good agreement with the results already discussed based on the analysis of dQ/dE and could indicate the formation of a more stable SEI for the PEDOT:PSSTFSI‐based composite electrode (Figures S21–S23, Supporting Information). In any case, further investigation of SEI formation during cycling should be carried out, and is in progress in our laboratories in order to get more insight into the composition of the SEI, its dynamics and stability.

For comparison, to assess the significant difference between PVDF and PEDOT:PSSTFSI polymers, long‐range cyclability tests were carried out for composite electrode with LFMP46 as active material and PVDF as binder, but without the addition of carbon black. Electrochemical performance was really poor, with a discharge capacity limited to 83.4 mAh g^−1^ for the first cycle at C/5 and an extremely rapid loss of capacity, as shown in Figure S24 (Supporting Information). A very high impedance is observed, with a resistance of 2000 Ω for the first cycle that increases drastically upon cycling up to 35 000 Ω at the end of the 150th cycle (Figure S25, Supporting Information). All these phenomena are due to limited electronic conductivity inside the electrode, which does not allow the carbon‐coated LFMP46 electrode to operate properly as motion of the electrons and Li^+^ ions are strongly correlated. At the local scale, in order to achieve a homogeneous intercalation of lithium, regardless the C‐rate, a charge compensation has to be achieved and requires fast enough electrons mobility all over the electrode.

## 3 Conclusion and Perspective

In this study, the use of PEDOT:PSSTFSI as an effective binder and conductive additive, replacing PVDF and carbon black used in conventional electrode for Li‐ion battery application, was demonstrated using commercial carbon‐coated LiFe_0.4_Mn_0.6_PO_4_ as positive electrode material. With its superior electrical and ionic conductivity, the complex PEDOT:PSSTFSI polymer has a beneficial impact on enhancing lithium diffusion within the electrode, increasing the reversible capacity at high rates, especially with high active material loading and improving the capacity retention upon long‐term cyclability. This development of new polymers for battery application is of high interest and promising for innovation in the field of lithium‐ion batteries in a near future, and of all‐solid‐state batteries in the mid‐term future. Indeed, any possibility to improve transport properties within thick electrodes and mechanical properties at the solid‐solid interfaces, as well as to mitigate detrimental reactivity at the interfaces, by removing for instance the carbon additive, will benefit to the technology. That type of polymers being mixed ionic and electronic conductors, can clearly be key players, despite a better understanding of the expansion behaviors and interface formation during cycling of the PEDOT:PSSTFSI composite electrode is still required.

## 4 Experimental Section

### Monomer Synthesis Potassium (4‐styrenesulfonyl)(trifluoromethylsulfonyl)imide, SSTFSIK

The SSTFSIK monomer was synthesized following procedure as described in the literature by Armand^[^ ^32^, ^35^ ^]^ to introduce the TFSI functional group that provides ionic conduction. The precursor used was 4‐styrenesulfonic acid sodium salt and the reaction were performed as described in Figure S1 (Supporting Information), in 160 mL of dry acetonitrile (Sigma–Aldrich), 8.0 mL of oxalyl chloride 98% (TCI) and 0.348 g extra dry N, N‐dimethylformamide (DMF, Sigma–Aldrich) and under stirring under argon atmosphere at room temperature. When the solution turned yellow, 16 g of 4‐styrenesulfonic acid sodium salt (Sigma–Aldrich) were added slowly to the solution, which was then stirred overnight. NaCl precipitate was removed by filtration, while 11.6 g of Trifluoromethanesulfonimide (TCI) were added in the filtrate solution, under stirring at 0 °C. Then, 32 mL triethylamine (Thermo Fisher) were added dropwise and the reaction was let for 16 h at room temperature. The precipitate was removed by filtration, the solvent was evaporated from the filtrate and the resulting brown solid was dissolved in 50 mL of dichloromethane. This solution was washed with 2 × 200 mL of an aqueous solution of NaHCO_3_ 4% (Sigma–Aldrich) and 100 mL of hydrochloric acid 1 m (Sigma–Aldrich). The potassium form of 4‐styrenesulfonyl(trifluoromethylsulfonyl)imide was obtained by neutralization of the acid monomer by a molar excess of K_2_CO_3_ (Sigma–Aldrich) in water. The resulting suspension was stirred for overnight, filtered, and dried to obtain a light‐yellow solid that was characterized with H‐NMR, F‐NMR, and C‐NMR in 1 mg mL^−1^ solution of deuterated DMSO using Bruker 400 Avance (Figure S3, Supporting Information).

### PSSTFSIK Synthesis

The polymerization of poly(4‐styrenesulfonyl(trifluoromethylsulfonyl)imide) potassium was done through reversible addition−fragmentation chain‐transfer (RAFT). To target a molecular weight of 100.000 g mol^−1^, 4.1 g of potassium 4‐styrenesulfonyl (trifluoromethylsulfonyl), 0.00038 g 2,2′‐Azobis(2‐methylpropionitrile (AIBN) (Sigma–Aldrich) and 0.00427 g 2‐(Dodecylthiocarbonothioylthio)‐2‐methyl propanoic acid (TCI) were added into a Schlenk line. Argon atmosphere was introduced before the addition of 10 mL of previously cryogenic‐distilled DMF (Sigma–Aldrich). The oxygen removal step by freeze‐thaw the solution in liquid nitrogen was performed three times. Solution was rigorously stirred at 65 °C for 24 h to let the polymerization reaction occur. Obtained viscous polymer solution was precipitated in diethyl ether for two times. The polymer was dried at 60 °C under vacuum for ≈24 h in order to remove the remaining solvents.^[^ ^32^, ^36^ ^]^ The molecular weight of polymer was analyzed by Size Exclusion Chromatography (SEC) in dimethyformamide (DMF + LiBr 1g L^−1^) as the eluent on an Ultimate 3000 system from Thermoscientific equipped with diode array detector (DAD), multi‐angles light scattering detector (MALS) and differential refractive index detector (dRI) from Wyatt technology.

Thermal analysis was done using Thermogravimetric Analysis (TGA) and Differential Scanning Calorimetry (DSC). Both analyses were carried out for PSSTFSIK and the dried PEDOT:PSSTFSI. Thermogravimetric Analysis (TGA) was done using TGA Q500 apparatus from TA instrument with a sample amount ≈4–6 mg under a nitrogen flow of 40–60 mL min^−1^, from room temperature to 800 °C and at a heating rate of 10 °C min^−1^. Differential Scanning Calorimetry (DSC) analysis was performed to examine the glass transition of the synthesized polymer, and using a TA instrument DSC Q200 LN2. 10–12.5 mg of the samples were measured in aluminum pans within the temperature range from −20 to 300 °C for PSSTFSIK and 0 to 180 °C for dried PEDOT:PSSTFSI using a heating and cooling rate varying from 10, 15, 20, and 25 °C min^−1^ for 2 cycles at each heating rate.

### PEDOT:PSSTFSI Synthesis

The PEDOT:PSSTFSI) complex was synthesized by classical oxidative polymerization of 3,4‐Ethylenedioxythiophene (EDOT) in the aqueous solution of the obtained PSSTFSI in deionized (DI) water as described in the literature.^[^ ^33^, ^34^ ^]^ The ratio of EDOT:SSTFSI was 0.5, while the molar ratio of (NH_4_)_2_S_2_O_8_:FeCl_3_ was 3.5, and oxidant ratio to EDOT monomer was 2.3. In round bottom flask, 190 µL EDOT were added to 1.025 g PSSTFSIK in 112.5 mL DI water solution and were vigorously stirred under a nitrogen atmosphere. Further, 6.25 mL of each 64 mg mL^−1^ (NH_4_)_2_S_2_O_8_ 98% (Sigma–Aldrich) and 13.6 mg mL^−1^ anhydrous FeCl_3_ (Sigma–Aldrich) were added as oxidants. After 72 h at 10 °C, the polymer dispersions were purified using Lewatit S100 KR/H and Lewatit MP62WS ion exchange resins (60 mg of resin per 1 mL of ink). The solution was then concentrated using an ultrafiltration cell with 100 kDa ultrafiltration discs (Amicon bioseparations) until maximum water could be removed and dark blue gel obtained.

### PEDOT:PSSTFSI Water Removal

Water in PEDOT:PSSTFSI was removed by freeze‐drying method as it can react with the battery active material and the electrolyte. 20 mL of the obtained dark blue gel was put in the 50 mL falcon, then frozen in liquid nitrogen and lyophilized using BenchTop Pro with Omnitronics with pressure of 75 µB and dew point of −80 °C for 2–3 days. Dark blue flaky powder was obtained and analyzed by TGA to determine its water quantity.

### Electrochemical Testing

The application of PEDOT:PSSTFSI as binder and electronic additive agent was done following standard slurry electrode preparation in N‐methyl‐2‐pyrrolidone (NMP). The commercial active material of carbon‐coated LiFe_0.4_Mn_0.6_PO_4_ (LFMP46 from S4R) was used as positive electrode material.

The dried PEDOT:PSSTFSI was dissolved in N‐methyl‐2‐pyrrolidone (NMP, Sigma–Aldrich) solvent for overnight at room temperature, the respective amount of active material was then added and stirred for 2 h minimum. For reference, active material was also ground together with carbon black (CB, Alfa Aesar) before being mixed with poly(vinylidene fluoride) (PVDF, Sigma–Aldrich) in NMP solution with mass ratio of 1:1 CB:PVDF. Slurry was casted on an aluminum current collector using a doctor blade with 200 µm thickness and dried at 80 °C for overnight. The casted electrode was cut with 16 mm diameter cutters, which were then calendared at 5 tons using pellet die, weighted, and vacuum dried overnight at 80 °C. The average mass loading of the electrode was 3.5–4 mg cm^−2^. After drying, the positive electrodes were transferred into an Argon filled glovebox (< 0.1 ppm oxygen and −75 °C dew point) for coin cells’ assembly. Four different active material (AM) percentages were investigated, i.e., 80 wt.% AM, 85 wt.% AM, 90 wt.% AM and 94 wt.% AM.

The electrochemical performance was tested in CR2032‐type coin cells. Half‐cells were assembled in Argon filled glovebox using lithium metal at the negative electrode and as reference, Whatman as separators, and commercial LP30 (1M LiPF_6_ in 1:1 v:v EC:DMC, from Solvionic) as electrolyte. Before any electrochemical test, the cells were allowed to rest for 6 h at temperature‐controlled room at 25 ^°^C. The assembled cells were tested with 3 different programs, i.e., 1) rate capability test by doing GCPL (Galvanostatic cycling with potential limitation) between 2.5 and 4.5 V vs Li^+^/Li with various C rates from C/10 to 4C, 2) long‐term cycling tests at C/5 for 150 cycles and 3) cyclic voltammetry with various scan rates (0.05, 0.1, 0.2, 0.3, 0.4, and 0.5 mV s^−1^). A rate of 1C corresponds to a current density to theoretically exchange 1 Li^+^ or 1 electron in 1 h per formula unit (full charge in 1 h).

Electrochemical impedance spectroscopy (EIS) analyses were performed to analyze the stability of the composite electrode during cycling and of the reactions occurring at the interfaces. Impedance measurements were performed in potential electrochemical impedance spectroscopy (PEIS) mode using BioLogic BT‐Lab Potentiostats and half‐cells assembled with 85 wt.% LFMP46 containing composite electrodes. The measurement was carried out from 10 kHz to 10 mHz at 25 ^°^C (C/5 for 150 cycles) before cycling, and at the end of the 1st cycle, 5th cycle, 25th cycle, 50th cycle, 100th cycle, and 150th cycle. The battery was rested for 6 h before each impedance measurement.

## Conflict of Interest

The authors declare no conflict of interest.

## Supporting information



Supporting Information

## Data Availability

The data that support the findings of this study are available from the corresponding author upon reasonable request.
