# Supplementary material for: An Alternative Polymer Material to PVDF Binder and Carbon Additive in Li‐Ion Battery Positive Electrode
Source: Adv Sci (Weinh). 2024 Oct 21;11(46):2409403. doi: 10.1002/advs.202409403 (PMC11633511; doi:10.1002/advs.202409403)
Supplement: Supplementary file 1 — Supporting Information [file ADVS-11-2409403-s001.docx]

Supporting Information

An Alternative Polymer Material to PVDF Binder and Carbon Additive in Li-Ion Battery Positive Electrode

Ivone Marselina NUGRAHA, Jacob OLCHOWKA, Cyril BROCHON,

Delphine FLAHAUT, Mélanie BOUSQUET, Benjamin CABANNES-BOUE, Rafael BIANCHINI NUERNBERG, Éric CLOUTET *, Laurence CROGUENNEC *

1. **METHODOLOGY**


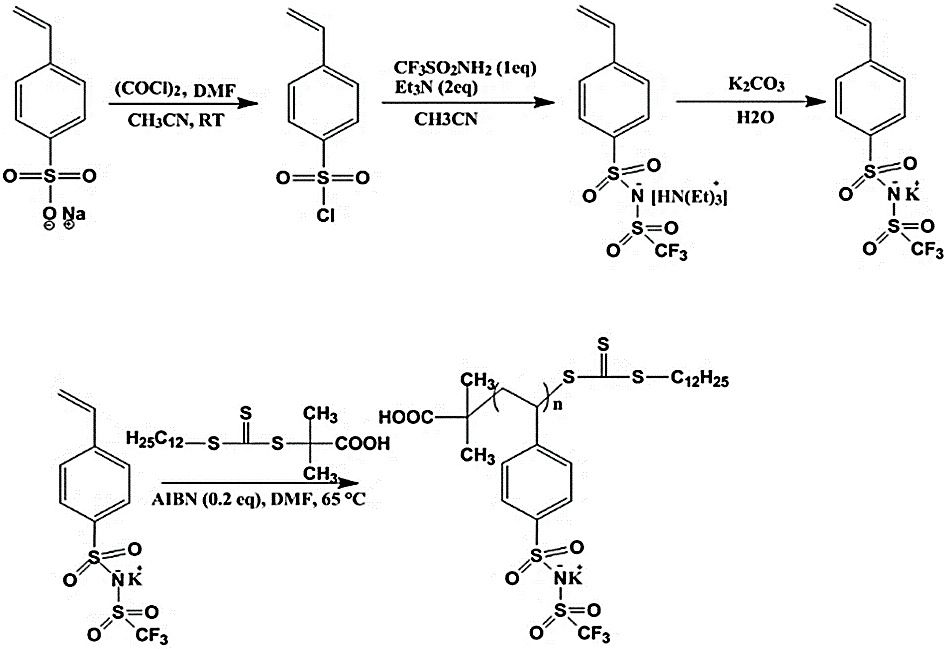


**Figure S1.** Synthesis of Monomer Potassium (4-styrenesulfonyl)(trifluoromethylsulfonyl)imide, SSTFSIK step

1. **CHARACTERIZATION**
   1. **X-ray photoelectron spectroscopy (XPS) characterization**

XPS analyses were carried out to understand the chemistry and interactions in the complex PEDOT:PSSTFSI. These analyses were done using a Thermo K-alpha spectrometer using a monochromatized Al-Kα X-Ray source (hν = 1486.6 eV) with an average analysis depth of 50 nm. The peaks are recording with following conditions: pass energy of 20 eV and an energy step of 0.1 eV with the analysis area of 200 x 400 µm². A neutralizer gun is used to minimize the surface charging and therefore charge compensation. The data analysis, quantification and spectra fitting were carried out using CASA XPS software. For the pristine powders, the spectral calibration was carried out using the CF_3_ component (292.3 eV) originated from the PSSTFSI chain in C1s core peaks. The peak fitting process was carried out using a non-linear Shirley-type background.

- 1. **Electrical conductivity measurement**

The electrical conductivity of the polymer was analyzed by the 4-probe method of bare casted film and its thickness by Dektak XT. Prior to the film deposition, 5 wt.% of DMSO and 0.05 wt.% of Zonyl FS300 fluoro-surfactant were added to all the PEDOT:PSSTFSI inks and stirred vigorously for overnight, to improve the film formation. The films were deposited on a previously cleaned glass substrate under ambient conditions using a doctor blade with 3 different gaps of 50, 75 and 100 µm and casting speed of 10 mm/s. The casted films were dried on a hot plate under air at 120^°^C for 5 minutes^[1]^.

The resistance measurement was done at least on 5 points for each casted film, while the Dektak XT was measuring the thickness of scratch and non-scratch parts of the film for at least 6 different points. The measured resistances and thickness values were then used to determine the electrical conductivity following this equation:

$\sigma=\frac{1}{R\times G\times t}$

Where $\sigma$ is the electrical conductivity value (S/cm), R is the measured resistance (Ω), and t is thickness of the film (cm). Meanwhile G is a geometry factor, which the value depends on the thickness of the film and the distance of probe (s, cm). As in this measurement, the distance of each probe (s) is 1.6 mm and the thickness of the film is in order of 500 – 5000 nm, thus it’s included as thin film samples (t <<< s) which the geometry factor follows this equations^[2,3]^:

$G=\frac{\pi}{ln2}=4.53$

- 1. **Ionic conductivity measurement**

The ionic conductivity was measured through impedance spectroscopy on drop-casted PEDOT:PSSTFSI films^[4]^. Similar to the solution preparation for electrical conductivity measurement, prior to the drop-casting, 5% wt.% of DMSO and 0.05 wt.% of Zonyl FS300 fluoro-surfactant were added to the PEDOT:PSSTFSI aqueous ink and stirred vigorously overnight to guarantee homogeneity. The films are produced by dropping 50 µL of the ink on a 9 mm^2^ masked area of conductive fluorine-doped tin oxide (FTO) glass electrode pieces and dried at room temperature overnight.

The impedance measurement was done in 2 electrodes cell setup. Sample electrode was placed in an open electrochemical cell as working electrode containing 0.5M LiClO_4_ in propylene carbonate mimicking the liquid electrolyte in Li-ion battery, while an epoxy-sealed platinum foil is used as reference/counter electrode. The cell was connected to BioLogic VMP-3e Multichannel Potentiostat impedance channel, and was oscillated with a 10 mV AC vs open circuit potential from 10^5^–10^-1^ Hz at 25^°^C. The ionic resistance was deduced from the width of the 45° high-frequency region of the resulting Nyquist plots following this equation:

$$\sigma_{ionic}=\frac{t}{3((Z_{real\left( low frequencies \right)}{-Z}_{real\left( high frequencies \right)})\times A)}$$

Where 𝜎_𝑖𝑜𝑛𝑖𝑐_ is the ionic conductivity value (S/cm), 𝑍_𝑟𝑒𝑎𝑙(𝑙𝑜𝑤 𝑓𝑟𝑒𝑞𝑢𝑒𝑛𝑐𝑖𝑒𝑠)_ the extrapolated low frequency intercepts (Ω), 𝑍_𝑟𝑒𝑎𝑙(ℎ𝑖𝑔ℎ 𝑓𝑟𝑒𝑞𝑢𝑒𝑛𝑐𝑖𝑒𝑠)_ high frequency intercepts (Ω), *t* the thickness of the sample (cm) and *A* the area. This equation is derived based on the model reported by Albery *et al.* for the A.C. impedance response of a film behaving like a finite transmission line^[5,6]^, with the assumption that the electronic resistance is negligible compared to the ionic resistance.

- 1. **Nanomechanical analysis using AFM (Atomic force microscopy)**

The nanomechanical properties were measured using AFM (Atomic force microscopy) on glass-casted thin film^[7,8]^. Atomic force microscopy was performed using an AFM (Dimension Icon, Bruker, Germany) in tapping mode with antimony doped Si cantilevers model RTESPA-300-30 (spring constant 40 N/m, resonance frequency 300 kHz). Before nanomechanical measurements of the samples, the probe was calibrated with standard polystyrene film with parameter of peak force setpoint of 20 nN and nanoindentation of 1 – 2 nm resulting in a Young modulus of 3 GPa. With the same nanoindentation value of 1-2 nm, to maintain the same contact surface between the probe and the sample, the previously prepared thin film was analysed. The measurements were carried out on a small area (2 μm × 2 μm). The result was treated with Nanoscope Analysis software (Bruker).

- 1. **Swellability measurement**

Swellability of the PEDOT:PSSTFSI was done by measuring the electrolyte mass uptake after soaking the electrode in the electrolyte overnight^[9]^. The dried PEDOT:PSSTFSI was dissolved in N-methyl-2-pyrrolidone (NMP, Sigma Aldrich) solvent with 5 wt.% concentration for overnight at room temperature. The solution was casted on an aluminium current collector using doctor blade with 200 µm thickness and dried at 80^°^C for overnight. Then, 16 mm diameter parts were cut, calendared at 5 tons using pellet die, weighted, and vacuum dried overnight at 80°C. For comparison, films with 5 wt.% poly(vinylidene fluoride) (PVDF, Sigma Aldrich) in NMP and 1:1 mass ratio of PVDF and carbon black (CB, Alfa Aesar) with 5 wt.% concentration in NMP were prepared in the same manner.

The weighted casted electrodes were immersed in an excess of LP30 (1M LiPF_6_ in 1:1 v:v EC:DMC, from Solvionic) electrolyte inside of Argon filled glovebox (< 0.1 ppm oxygen and -75^0^C dew point) and left swell naturally for overnight. Afterwards, the excess electrolyte was absorbed using Kimwipe and its mass was measured.

- 1. **PEDOT:PSSTFSI stability test**

The stability of PEDOT:PSSTFSI was analysed to assess its reactivity with the liquid electrolyte and determine its stability window. Thus, cyclic voltammetry in potential range from 0-6 V vs Li^+^/Li with scan rates of 0.25, 0.5, 0.75, and 1 mV/s was performed. This cyclic voltammetry was done using half-cell CR2032-type coin cells vs lithium metal anode, using LP30 electrolyte and Whatman separator, as for the electrochemical tests performed in this study. Two types of battery have been assembled 1) with only aluminium current collector as cathode (noted as only electrolyte) and 2) with PEDOT:PSSTFSI electrode prepared similarly as the one for swellability measurement.

- 1. **X-ray diffraction (XRD) analysis**

X-ray diffraction (XRD) experiments were carried out with a laboratory X’Pert3 diffractometer equipped with a capillary spinner and a Cu Kα_1,2_ X-ray source. The powder was packed in a 0.5 mm diameter glass capillary and measured with steps of 0.016°.

- 1. **Scanning Electron Microscopy (SEM)**

The homogeneity of the electrode and the coating of polymer on active material in pristine and after cycling were analysed by scanning electron microscopy (SEM). SEM images were collected using a TESCAN Vega microscope operating at a 15 kV accelerating voltage with a tungsten source.

- 1. **Electrode Porosity**

The electrode porosity was analysed by following equation:

$$electrode porosity=\frac{(\rho_{\mathrm{theoretical}}-\rho_{\mathrm{experimental}})}{\rho_{\mathrm{theoretical}}}\times100\%$$

$$\rho_{\mathrm{theoretical}}=\frac{1}{\sum\frac{mass fraction of components}{\rho_{\mathrm{components}}}}$$

The $\rho_{\mathrm{components}}$ was analysed using Helium Pycnometer (Quantachrome: UltraPycnometer 1000) at room temperature with flow mode of 3 minutes for 100 times, the obtained value is an average for all measured values. While the $\rho_{\mathrm{experimental}}$ was calculated following this equation:

$$\rho_{\mathrm{experimental}}=\frac{mass of electrode (g)}{Area of electrode \left( {cm}^{2} \right)\times electrode thickness \left( cm \right)}$$

In this calculation, as electrode was cut with diameter of 16 mm, thus the area of electrode is 2.01 cm^2^. The thickness of each electrode was measured at least on five points using a digital micrometer. This measurement was then adjusted by subtracting the thickness of the aluminium current collector (18 µm).

1. **Additional results**
   1. **Monomer structural characterization (^1^H-NMR & ^19^F-NMR)**

The ^1^H-NMR, ^13^C-NMR and ^19^F-NMR spectra of synthesized SSTFSIK obtained in 1 mg/mL solution of deuterated DMSO are shown below.


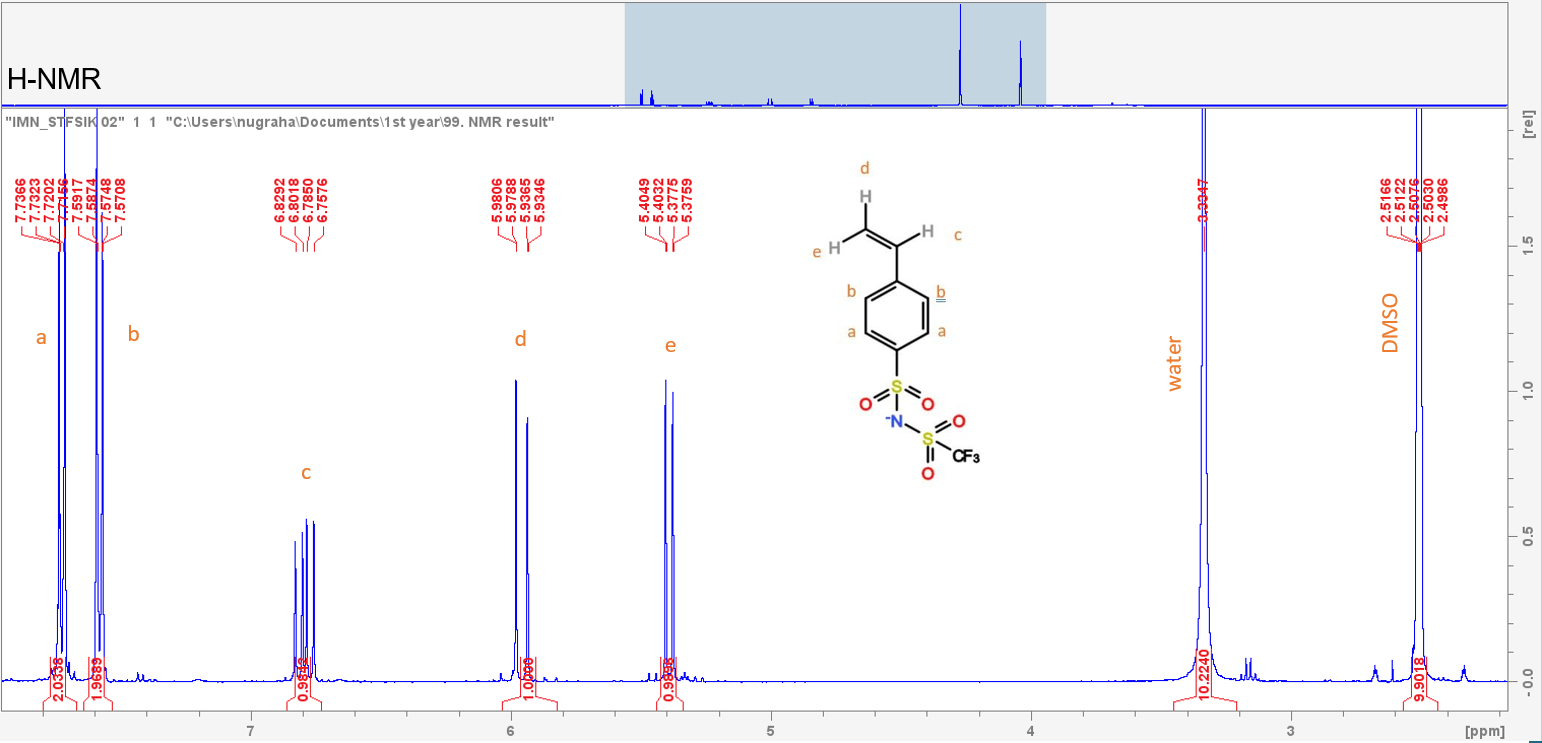


^1^H NMR (400 MHz, DMSO-d_6_, ppm): 5.38 (CH_2_=CH-, d, 1H), 5.95 (CH_2_=CH-, d, 1H), 6.77 (CH_2_=CH-, dd, 1H), 7.64 (C_6_H_4_-, dd, 4H).


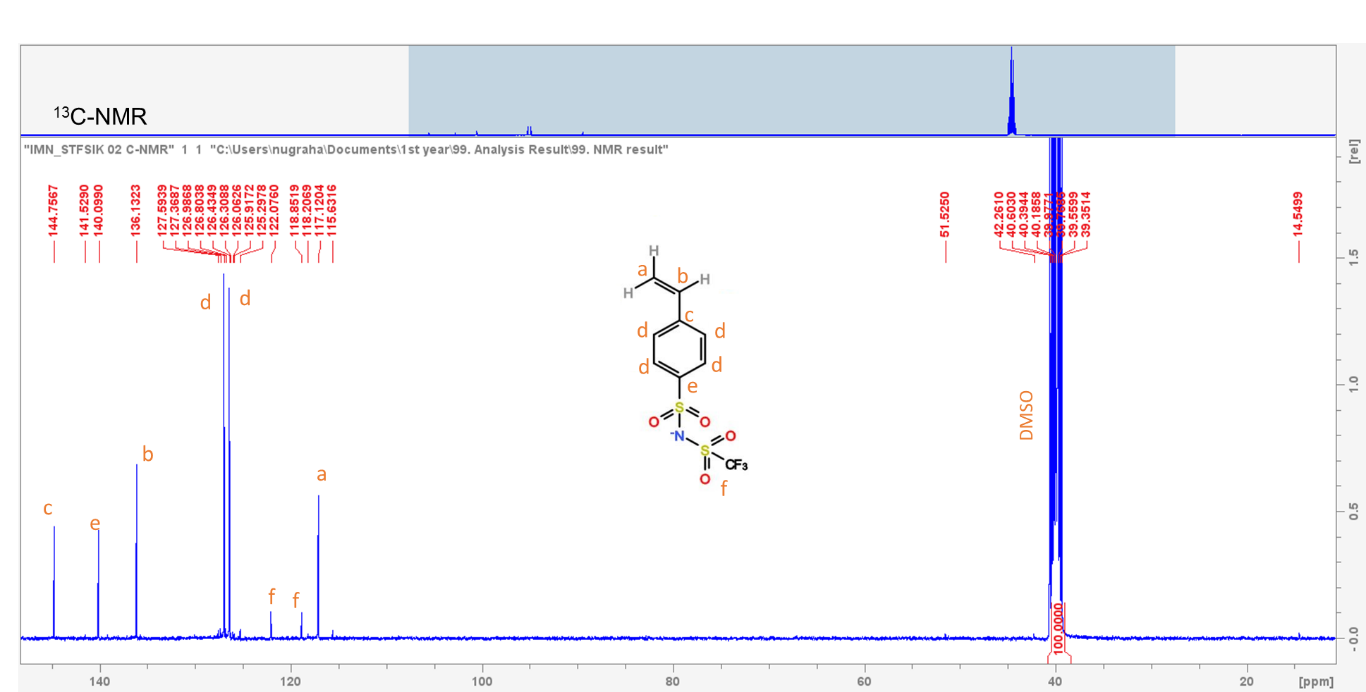


^13^C NMR (400 MHz, DMSO-d_6_, ppm): 116.78 (CH_2_=CH-), 118.48 and 121.70 (-CF_3_), 126.07-126.61 (-CH_2_ aromatic), 135.74 (CH_2_=CH-), 139.77 and 144.35 (-CH aromatic).


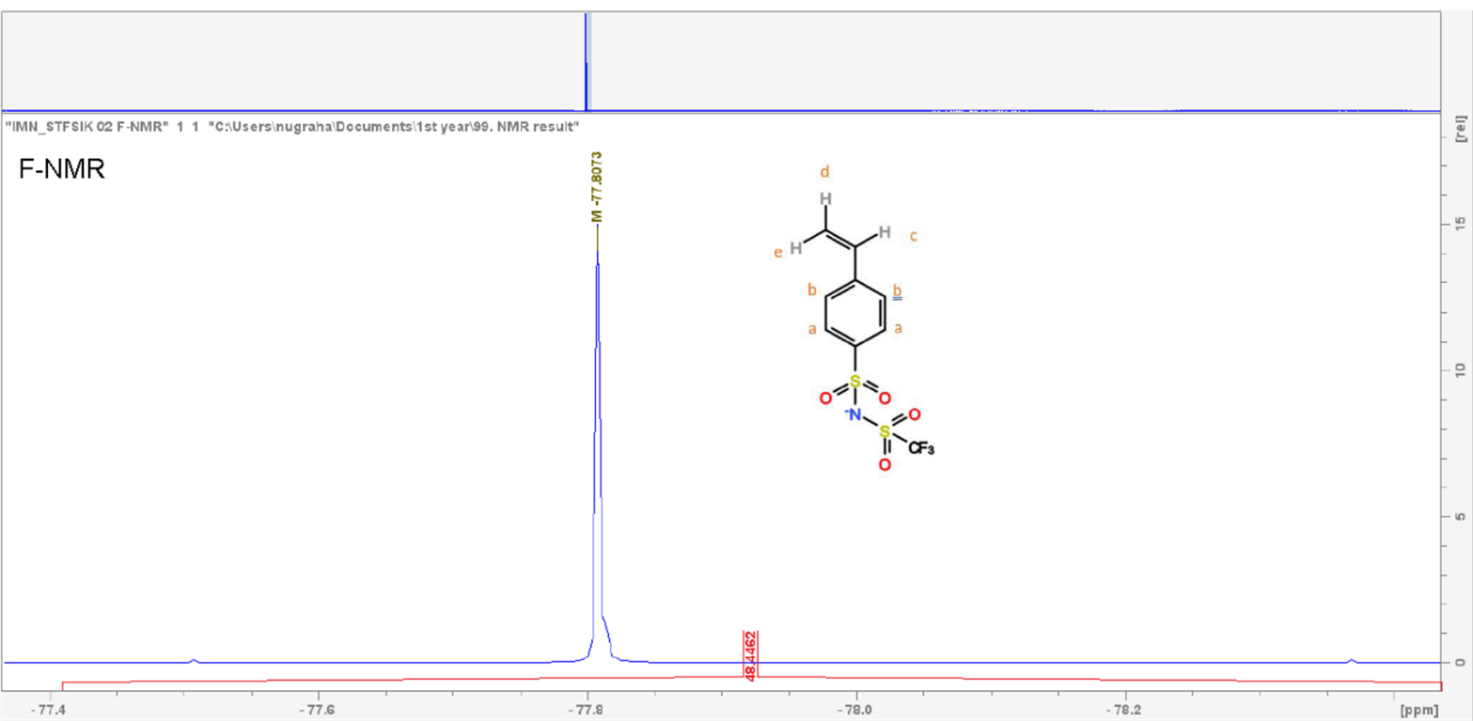


^19^F NMR (400 MHz, DMSO-d_6_, ppm): -77.81. 7 (-CF_3_, s, 3 F).

**Figure S2.** ^1^H-NMR, ^13^C-NMR and ^19^F-NMR of synthesized SSTFSIK in d_6_-DMSO

From these NMR spectra, all peaks are corresponding to pure phase of SSTFSIK, showing thus that the TFSI functionalization was successfully done on styrene-based monomer resulting in SSTFSIK monomer.

- 1. **SEC results**

The Size Exclusion Chromatography (SEC) in DMF trace for the synthesized polymer is presented hereafter:


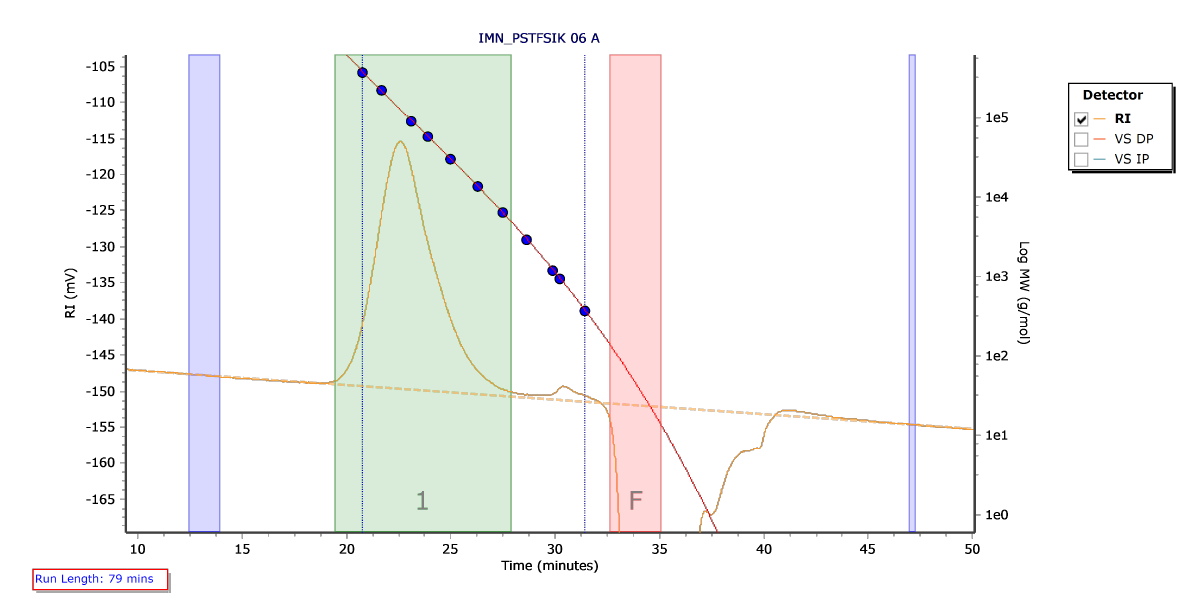


**Figure S3.** SEC traces for synthesized PSSTFSIK

**Table S1.** SEC results

| $\bar{\boldsymbol{M}}$**_p_ (g/mol)** | $\bar{\boldsymbol{M}}$**_n_ (g/mol)** | $\bar{\boldsymbol{M}}$**_w_(g/mol)** | **Đ** |
| --- | --- | --- | --- |
| 125000 | 64000 | 134000 | 2.1 |

Whereas:

Mp : molecular weight of the highest peak

Mn : number average molecular weight

Mw : weight average molecular weight

One main peak is observed in the SEC trace, corresponding to the PSSTFSIK polymer. The dispersity (Đ = Mw/Mn vs. PS) was calculated with a calibration curve based on narrow polystyrene (PS) standards, using only the RI detector.

- 1. **Thermal analysis Results**
     1. *PSSTFSIK*


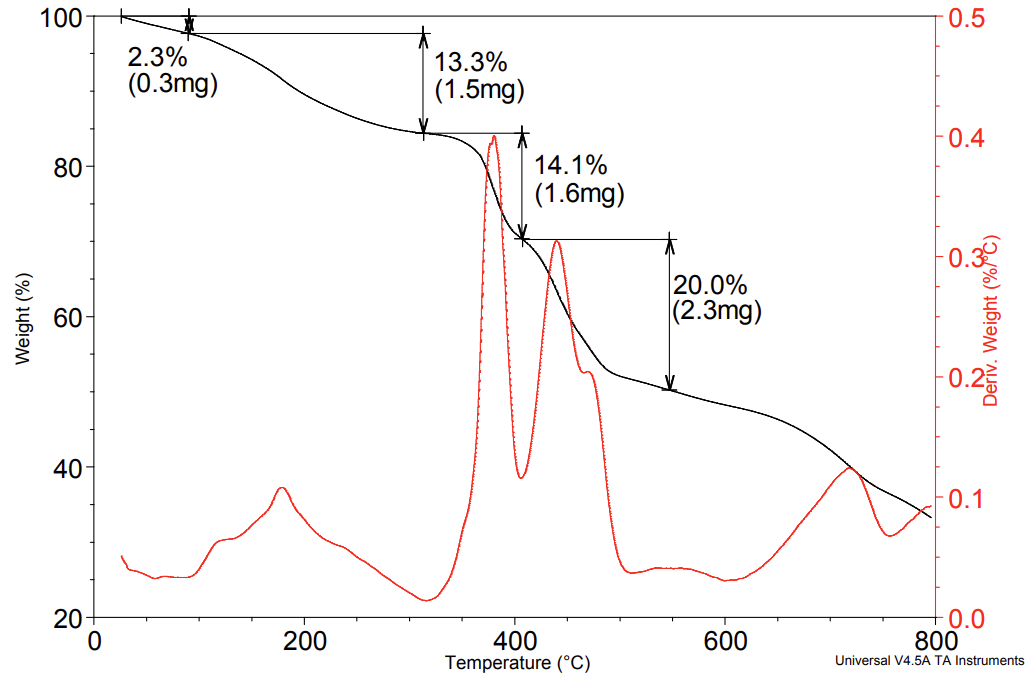


**Figure S4.** TGA result for PSSTFSIK

The TGA shows 3 domains: 1) below 320^°^C the weight loss is due to traces of DMF solvent trapped in PSSTFSIK polymer chain, 2) from 320°C to 500°C, a two-step weight loss is observed with the degradation of C-S bond in TFSI chain at 380^°^C and degradation of polystyrene backbone at 420^°^C and lastly 3) above 600°C, full degradation of PSSTFSIK. This TGA result is similar with the result reported by Meziane, R. *et al.*^[10]^.


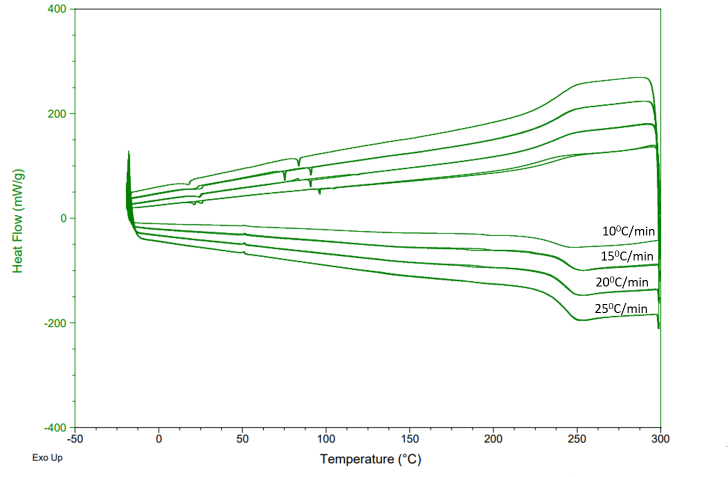

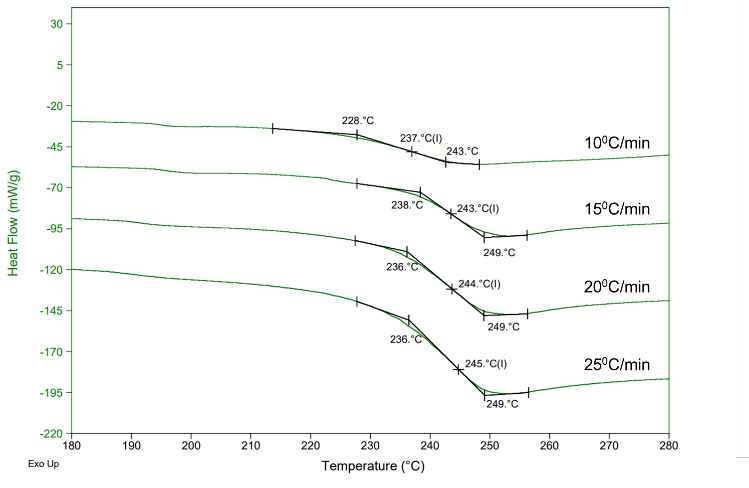


**Figure S5.** DSC results for PSSTFSIK

This DSC analyses reveal that PSSTFSIK is characterized by a glass transition temperature (Tg) of 237^°^C at the heating rate of 10^°^C/min, which is shifted to higher temperature as the heating rate increases.

- - 1. *PEDOT:PSSTFSI*

After freeze-drying, the water content of the complex PEDOT:PSSTFSI polymer was evaluated by TGA as shown in figure S6 below:


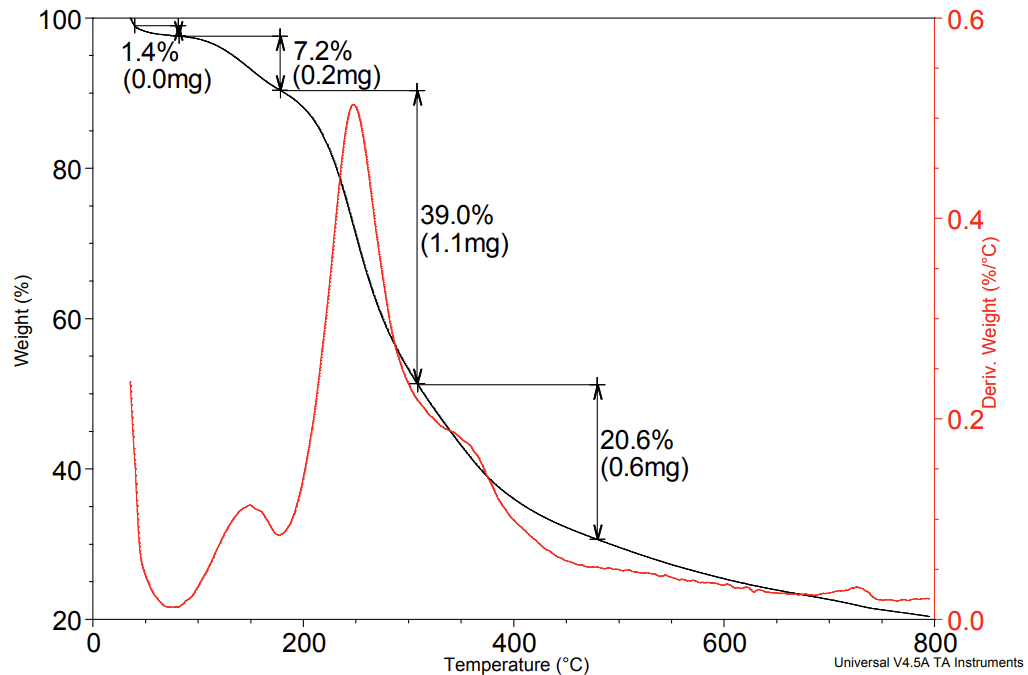


**Figure S6.** TGA result for freeze dried PEDOT:PSSTFSI

After freeze-drying, PEDOT:PSSTFSI shows a water content around 1.4 wt.%. This small content of residual water might be due to hydrogen bonding in the PEDOT:PSSTFSI complex. Anyhow, weight loss of 7.2 wt.% before 200^0^C for PEDOT:PSSTFSI is observed that might be caused by the traces of trapped DMF solvent from the synthesis of PSSTFSIK.


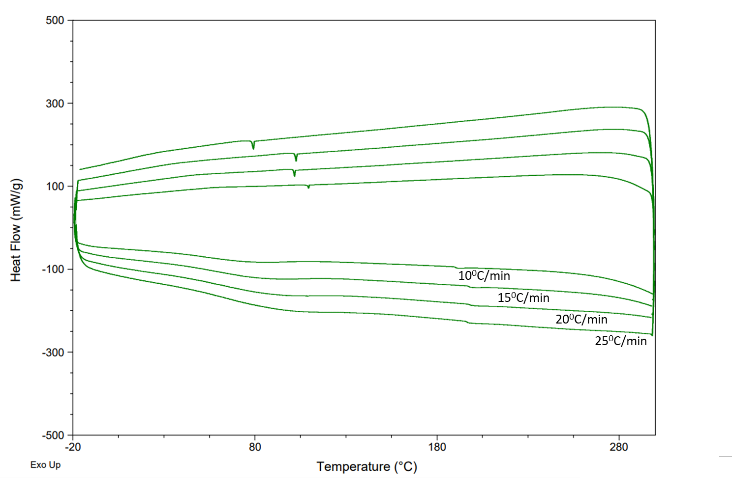

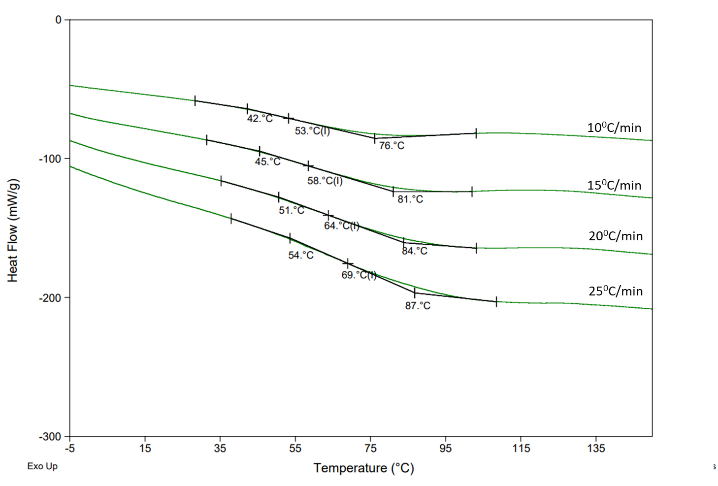


**Figure S7.** DSC results of PEDOT:PSSTFSI

The DSC results of dried complex PEDOT:PSSTFSI show a glass transition temperature at around 53^°^C with the heating rate of 10^°^C/min, and shifted to higher temperature as the heating rate increases.

- 1. **Additional XPS Result**
     1. *XPS survey spectra*

The result of XPS survey spectra for PSSTFSIK and PEDOT:PSSTFSI is shown as follow. Five corresponding main elements are detected for both samples, namely oxygen, carbon, nitrogen, fluorine, and sulfur. Meanwhile, the presence of potassium is detected for PSSTFSIK but not for PEDOT:PSSTFSI, confirming that the purification of synthesized PEDOT:PSSTFSI using ion exchange resin successfully removed the potassium from the system. Furthermore, no other elements/impurities are detected.


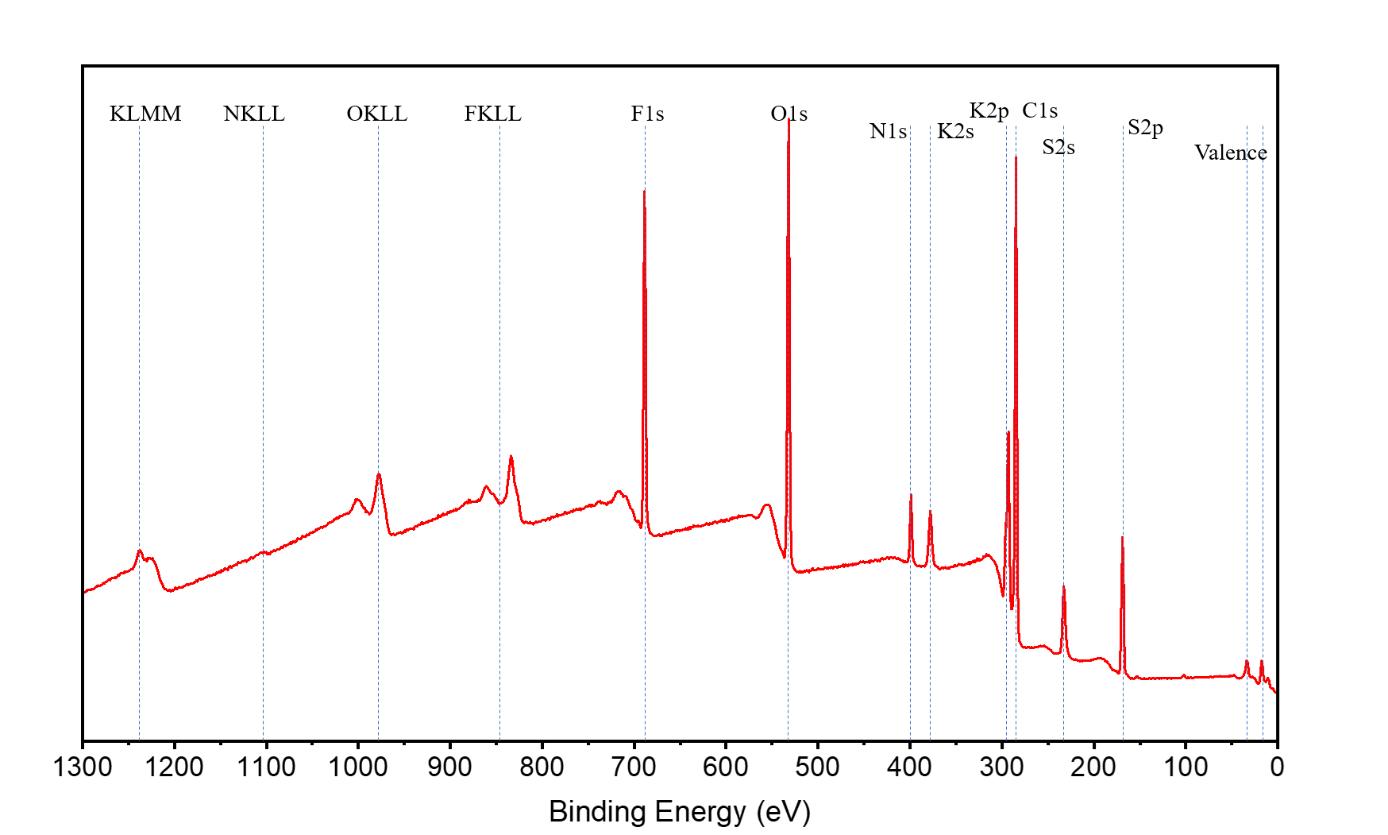


**Figure S8.** XPS Survey Result of PSSTFSIK


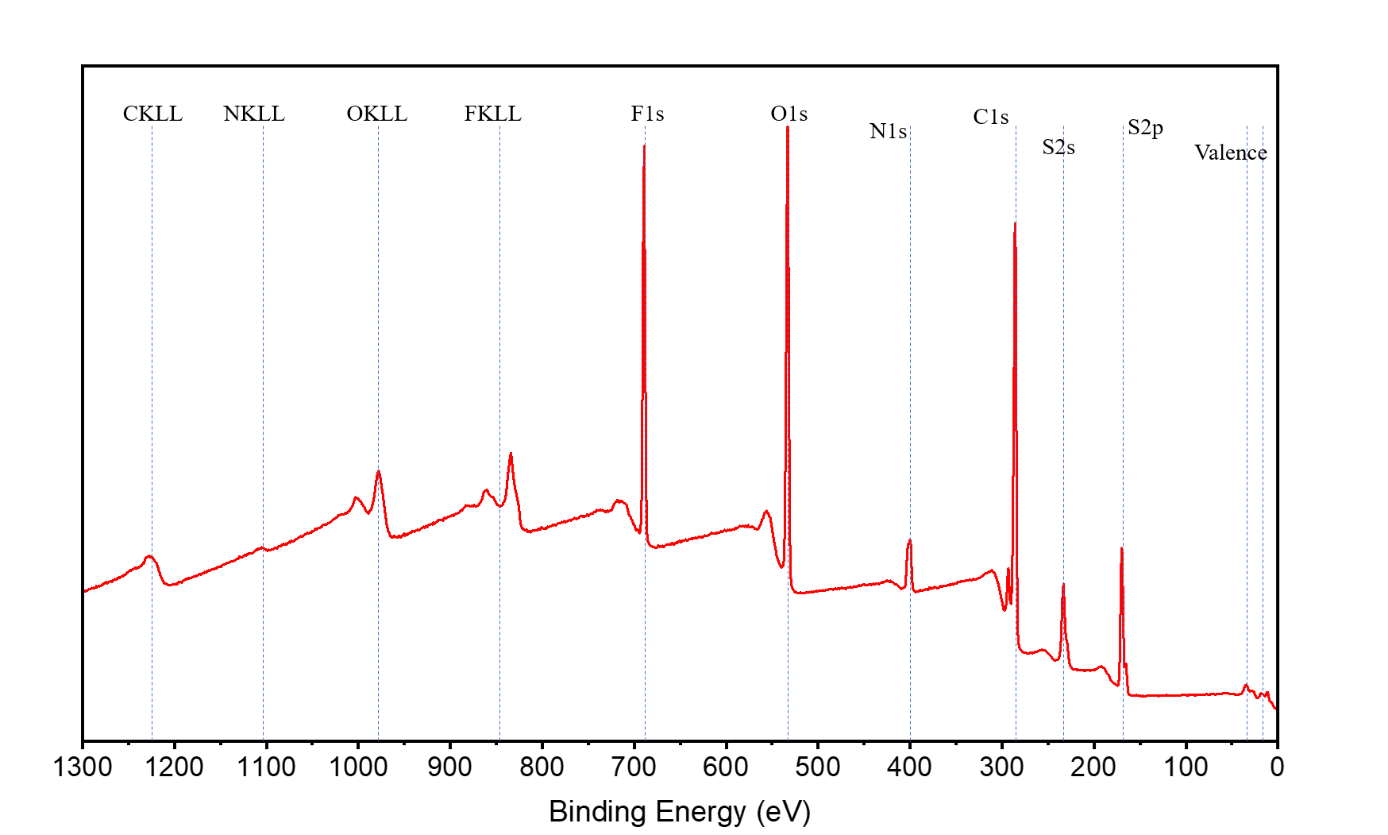


**Figure S9.** XPS Survey Result of complex PEDOT:PSSTFSI

- - 1. *N1s and S2p core spectra*

For understanding the binding properties of PSSTFSIK and the complexation of PEDOT:PSSTFSI, N1s core spectrum is used to determine the state of PSFTSI chain in the system. As shown in figure S10, 3 different states of PSSTFSI chain could be determined, i.e. 1) negatively charge PSSTFSI^-^ (398.4 eV), 2) neutral PSSTFSIH (400 eV), and 3) PSSTFSI interaction with hydrogen bonding (401.9 eV)^[1]^. Correspondingly, XPS spectra shows two N1s peaks for PSSTFSIK corresponding to neutral state of PSSTFSIH and negatively charge PSSTFSI^-^ that then forms ionic bonding with potassium ion. Meanwhile for complex PEDOT:PSSTFSI, the presence of water during the synthesis process induces the presence of hydrogen bonds in complex PEDOT:PSSTFSI.


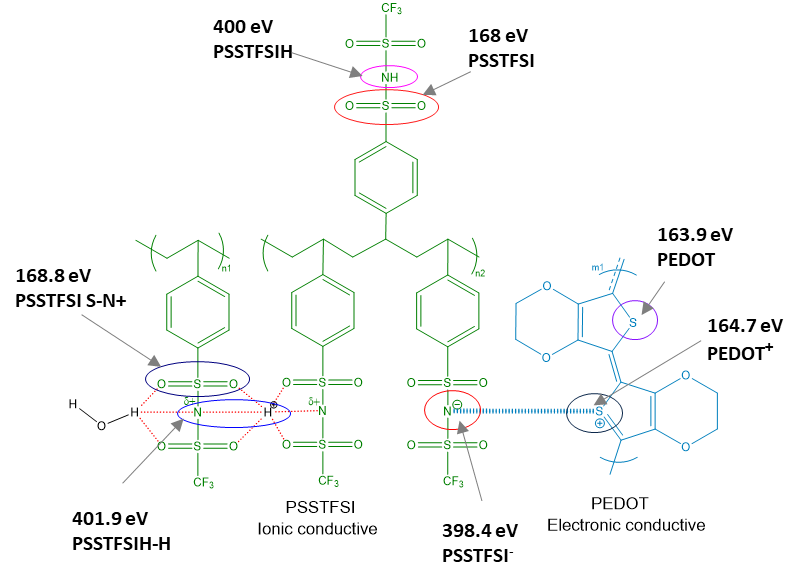


**Figure S10.** Complex PEDOT:PSSTFSI with the corresponding N1s and S2p core peak values from XPS analysis

- - 1. *XPS quantification results*

The quantification of the XPS fitting result is shown as follows:

**Table S2.** Binding energies (eV), FWHM values and atomic percentages for the PSSTFSIK

| **Orbitals** | **Binding Energy (eV)** | **FWHM** | **at.%** | **Assignments** |
| --- | --- | --- | --- | --- |
| C1s | 284.6 | 1.1 | 46.5 | C-C/C-H |
|  | 285.6 | 1.5 | 3.5 | O=C-N traces from DMF solvent |
|  | 288.5 | 1.2 | 0.4 | C=O traces from RAFT agent |
|  | 292.3 | 1.6 | 1.0 | CF_3_ of PSSTFSIK |
|  |  |  | **51.4** |  |
| K2p | 293.0 | 1.4 | **6.9** | Potassium in PSSTFSIK |
| F1s | 688.3 | 1.7 | **9.4** | CF_3_ of PSSTFSIK |
| O1s | 532.0 | 1.4 | 15.2 | O=S=O of PSSTFSIK |
|  | 530.5 | 1.4 | 1.5 | O=C-N traces from DMF solvent |
|  | 533.3 | 1.6 | 1.3 | H_2_O traces |
|  |  |  | **18.0** |  |
| N1s | 398.7 | 1.3 | 3.5 | PSSTFSIK (negatively charged PSFTSI- chain) |
|  | 400.0 | 1.4 | 0.5 | PSSTFSIH (neutrally charge PSTFSI chain) |
|  |  |  | **4.0** |  |
| S2p | 168.3 | 1.1 | 9.4 | O=S=O of PSSTFSIK |
|  | 165.8 | 1.0 | 0.4 | C=S traces from RAFT agent |
|  | 163.3 | 1.2 | 0.3 | C-S-C traces from RAFT agent |
|  | 166.8 | 1.1 | 0.1 | C-S-C traces from RAFT agent |
|  |  |  | **10.2** |  |

**Table S3.** Binding energies (eV), FWHM values and atomic percentages for the PEDOT:PSSTFSI

| **Orbitals** | **Binding Energy (eV)** | **FWHM** | **at.%** | **Assignments** |
| --- | --- | --- | --- | --- |
| C1s | 284.6 | 1.2 | 35.3 | C-C/C-H |
|  | 286.2 | 1.6 | 13.9 | C-O of PEDOT |
|  | 288.4 | 1.4 | 0.5 | C=O traces from RAFT agent |
|  |  |  |  |  |
|  | 292.3 | 1.8 | 4.3 | CF_3_ of PSSTFSI |
|  |  |  | **54.1** |  |
| F1s | 688.4 | 1.9 | 9.8 | CF3 of PSSTFSI |
|  |  |  | **9.8** |  |
| O1s | 531.8 | 1.5 | 10.1 | O=S=O of PSSTFSI |
|  | 533.0 | 1.6 | 7.1 | C-O of PEDOT |
|  | 534.4 | 1.7 | 0.9 |  |
|  |  |  | **18.1** |  |
| N1s | 398.4 | 1.2 | 1.9 | PSSTFSI^-^ (negatively charged PSFTSI- chain) |
|  | 400.0 | 1.8 | 2.1 | PSSTFSIH (neutrally charge PSSTFSI chain) |
|  | 401.9 | 1.3 | 1.6 | PSSTFSI S-N+ (complex with water) |
|  |  |  | **5.6** |  |
| S2p | 167.9 | 1.1 | 4.3 | O=S=O of PSSTFSI |
|  | 165.4 | 1.0 | 0.3 | C=S traces from RAFT agent |
|  | 162.9 | 1.2 | 0.1 | C-S-C traces from RAFT agent |
|  | 166.4 | 1.1 | 0.3 | C-S-C traces from RAFT agent |
|  | 168.7 | 1.5 | 5.4 | PSSTFSI S-N+ (complex with water) |
|  | 163.9 | 0.9 | 1.4 | Neutral PEDOT C-S-C |
|  | 164.8 | 1.3 | 0.6 | Positively charged PEDOT C-S-C |
|  |  |  | **12.4** |  |

- 1. **Electrical conductivity results**

The electrical conductivity was measured on doctor blade casted thin films and for each film 6 points were measured for both resistivity by 4-probe method and thickness by Dektak XT profilometer.

**Table S4.** Comparison of electrical conductivity results between PEDOT:PSSTFSI and 1:1 PVDF:CB thin films.

| **Item** | **PEDOT:PSSTFSI film made from an aqueous ink (before freeze-drying)** | | | **PEDOT:PSSTFSI film made from an NMP-based ink (after freeze-drying)** | | | **1:1 PVDF:CB**  **(5 wt.% in NMP)** | | |
| --- | --- | --- | --- | --- | --- | --- | --- | --- | --- |
| **Dr. Blade Gap** | **100µm** | **75µm** | **50µm** | **100µm** | **75µm** | **50µm** | **100µm** | **75µm** | **50µm** |
| Resistivity (Ω) | 118.9  ±8.3 | 121.9±36.1 | 211.3±31.4 | 81.3  ±8.4 | 100.4  ±14.0 | 76.2  ±22.2 | 539  ±133.0 | 495.6  ±59.0 | 189.9  ±9.3 |
| Thickness (nm) | 710.4  ±27.3 | 522.5±30.1 | 358.4±10.6 | 3781.2±93.6 | 3895.2±297.8 | 3831.8±371.5 | 3219.7  ±457 | 3955.7±792 | 4750.5±264 |
| Conductivity (S/cm) | 118.9±9.4 | 157.0±47.4 | 132.1±20.0 | 32.5±3.5 | 25.6±4.1 | 34.3±10.5 | 5.8±1.6 | 5.1±1.2 | 11.1±0.8 |
| Average (S/cm) | 135.8±17.43 | | | 30.8±3.93 | | | 7.3±0.73 | | |

A higher electrical conductivity is observed for the PEDOT:PSSTFSI film made in water and before freeze-drying (135.8±19.6 S/cm), the decrease of the electrical conductivity of PEDOT:PSSTFSI after freeze-drying might be due to the change of PEDOT:PSSTFSI chain which affects its total electrical conductivity.

- 1. **Ionic conductivity results**

The impedance spectrum of the ionic conductivity measurement is shown in the figure below:


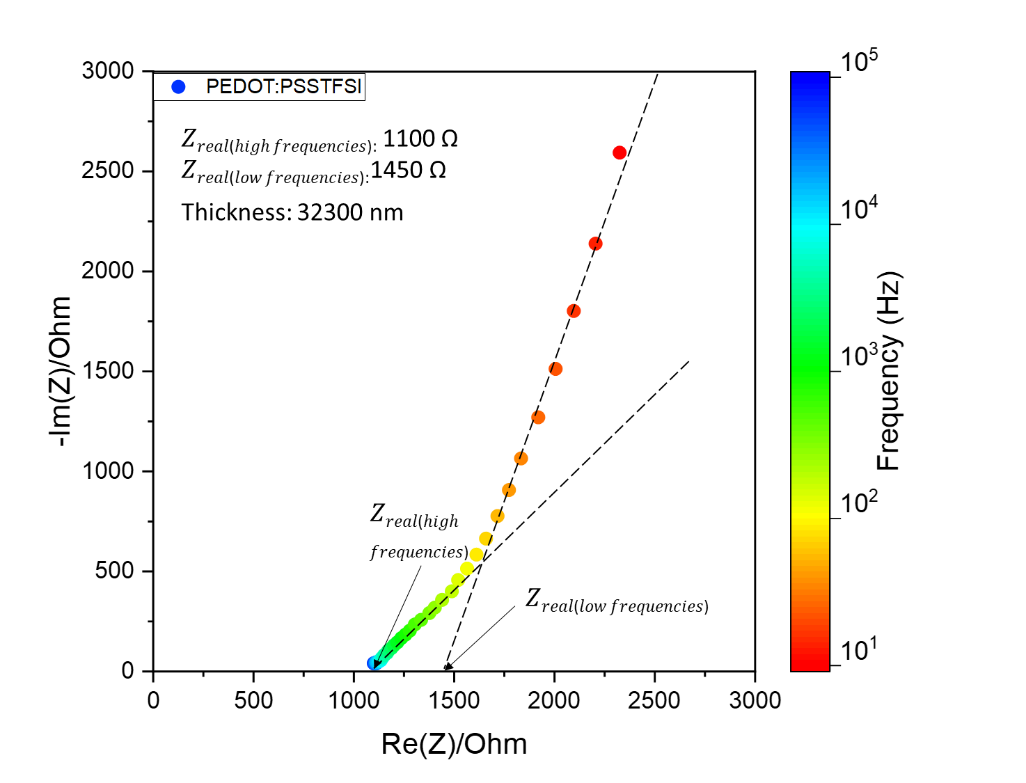


**Figure S11.** Nyquist plot of ionic conductivity measurement performed for PEDOT:PSSTFSI

Following the equation that has been already presented, an ionic conductivity of 3.4 10^-5^ S.cm^-1^ is obtained for PEDOT:PSSTFSI.

- 1. **Nanomechanical properties results**


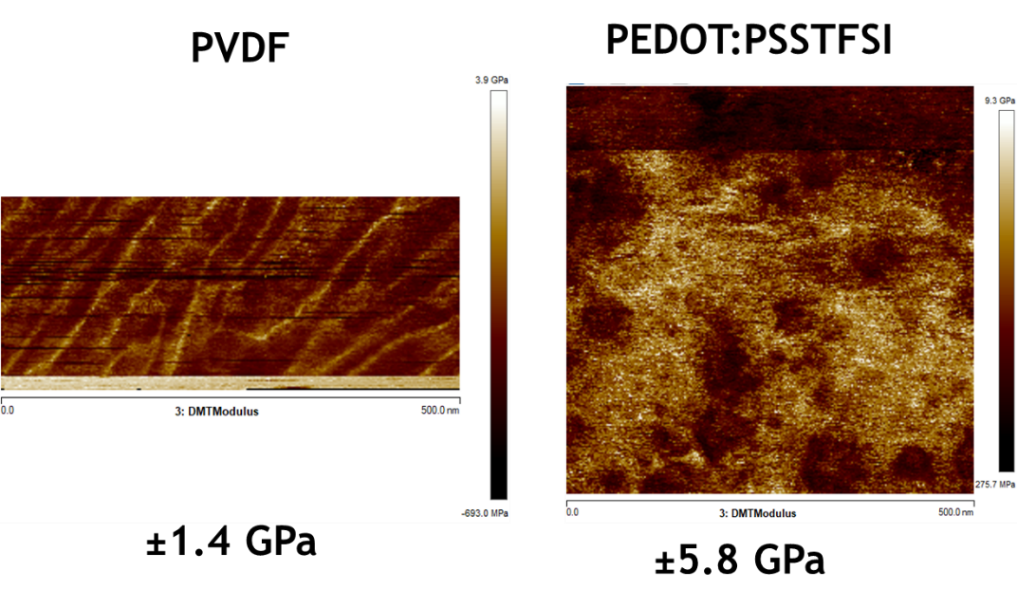


**Figure S12.** Young modulus results of PVDF and PEDOT:PSSTFSI films using AFM

*
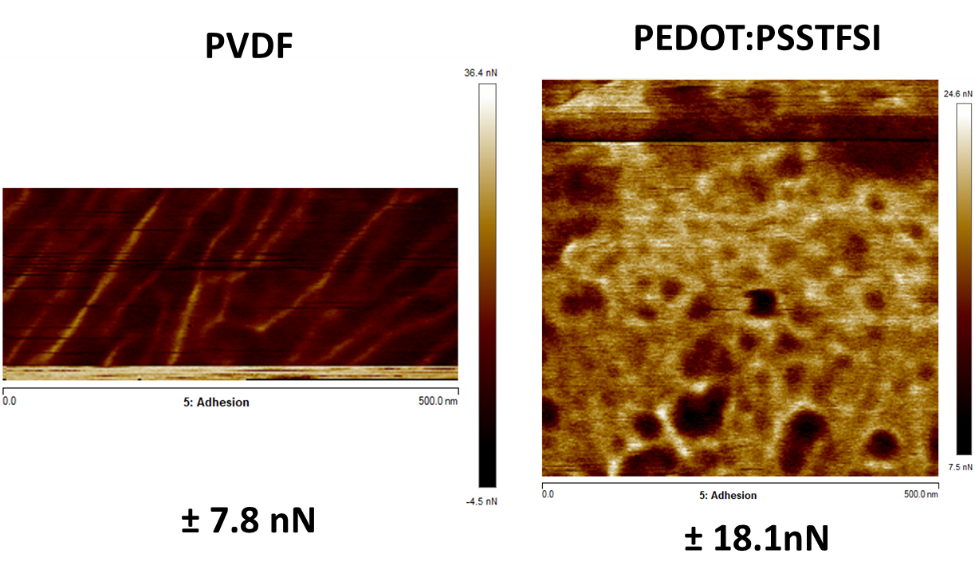
*

**Figure S13.** Results of adhesion forces of PVDF and PEDOT:PSSTFSI films using AFM

- 1. **Swellability measurement results**

The mass intake of swollen polymer is shown in this following figure.


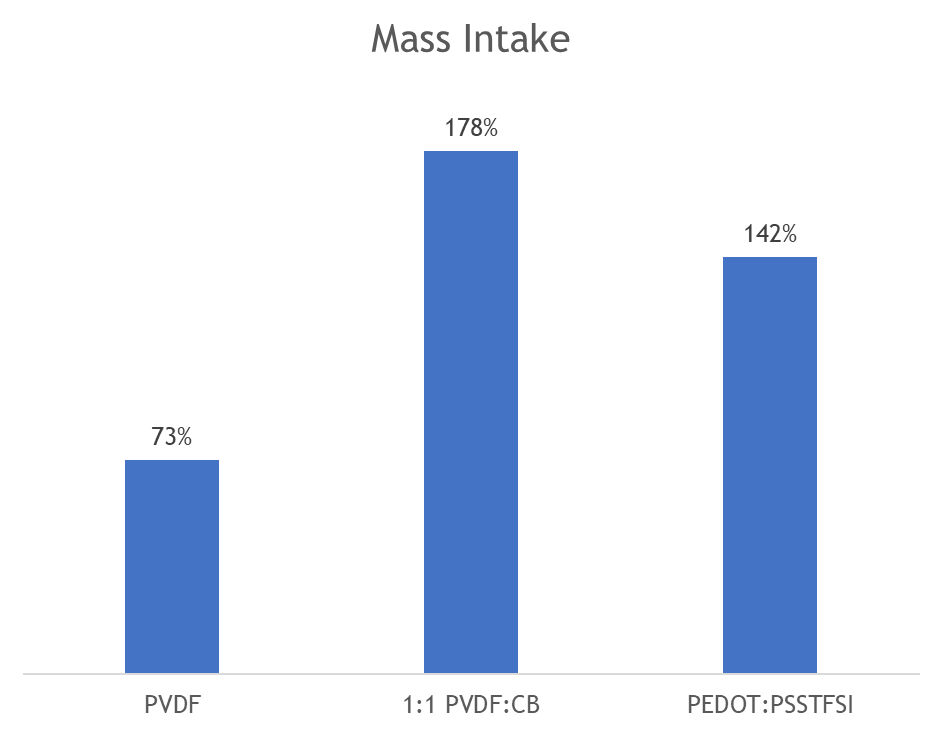


**Figure S14.** Mass intake for each polymer sample after immersion into a LP30 (1M LiPF_6_ in 1:1 v:v EC:DMC) electrolyte.

From the figure above, it is observed that PEDOT:PSSTFSI swells better than PVDF as a higher mass intake is measured. This phenomenon might be due to PEDOT:PSSTFSI polarity that helps the electrolyte to interact better with the polymer. The highest mass intake of 1:1 PVDF:CB is caused by high porosity of the carbon black that increase the immersion of electrolyte in the electrode through its pores.

- 1. **PEDOT:PSSTFSI stability test result**


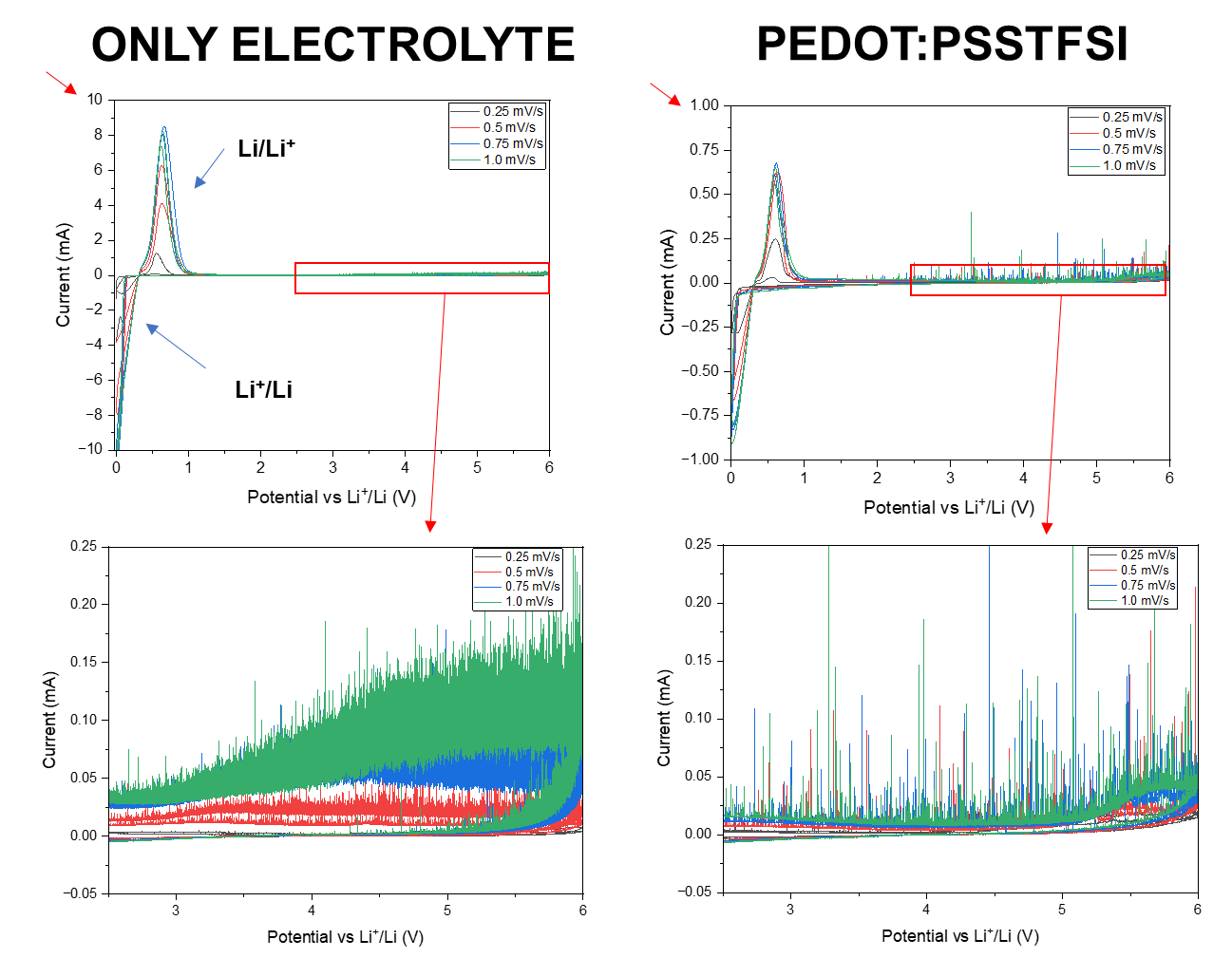


**Figure S15.** Cycling voltammogram for a) only electrolyte battery and b) PEDOT:PSSTFSI electrode battery.

- 1. **XRD results**

XRD has been performed to analyse potential change of the LFMP46 pristine material after composite electrode preparation. Moreover it could be used to analyse if there is any crystalline domain of PEDOT:PSSTFSI in the composite electrode.

**Figure S16.** XRD patterns of LFMP pristine and lab-scale LFMP46-PEDOT:PSSTFSI

All the observed peaks of LFMP-PEDOT:PSSTFSI correspond to those of the LFMP pristine powder. If we compare the XRD patterns of LFMP-PEDOT:PSSTFSI with the results obtained by Yousefian *et al.*, it appears that no extra peak are observed at 2θ of 12.4^°^ and 25.9^°[11]^, indicating that the PEDOT:PSSTFSI here is mostly amorphous.

- 1. **SEM image of electrode**

Prior to the electrochemical tests, SEM images of the electrode were done to analyze its homogeneity compared to the reference. It can be observed that the PEDOT:PSSTFSI composite electrode with LFMP46 shows satisfactory homogeneity compared to the reference electrode.

| 1. 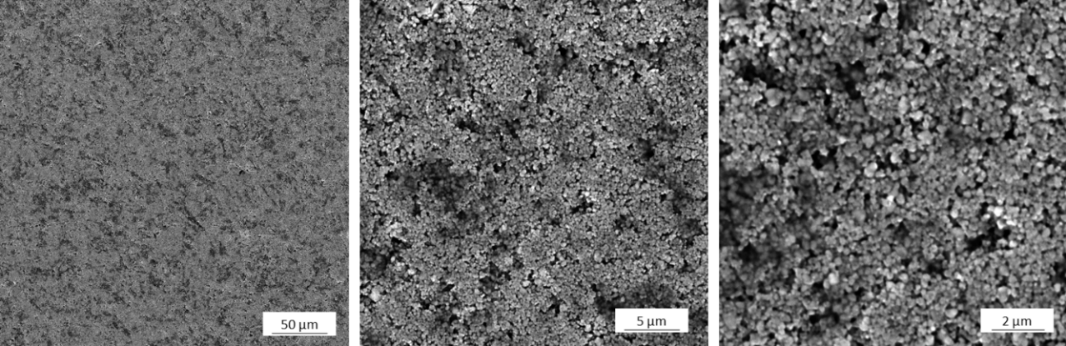 |
| --- |
| 1. 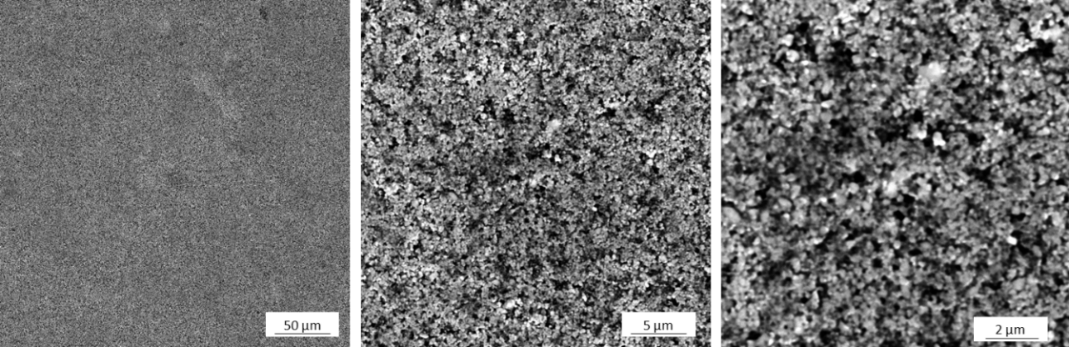 |

**Figure S17.** SEM images of pristine state a) 85 wt.% LFMP46-15 wt.% PEDOT:PSSTFSI and b) 85 wt.% LFMP46 - 15 wt.% PVDF:CB (1:1)

1.
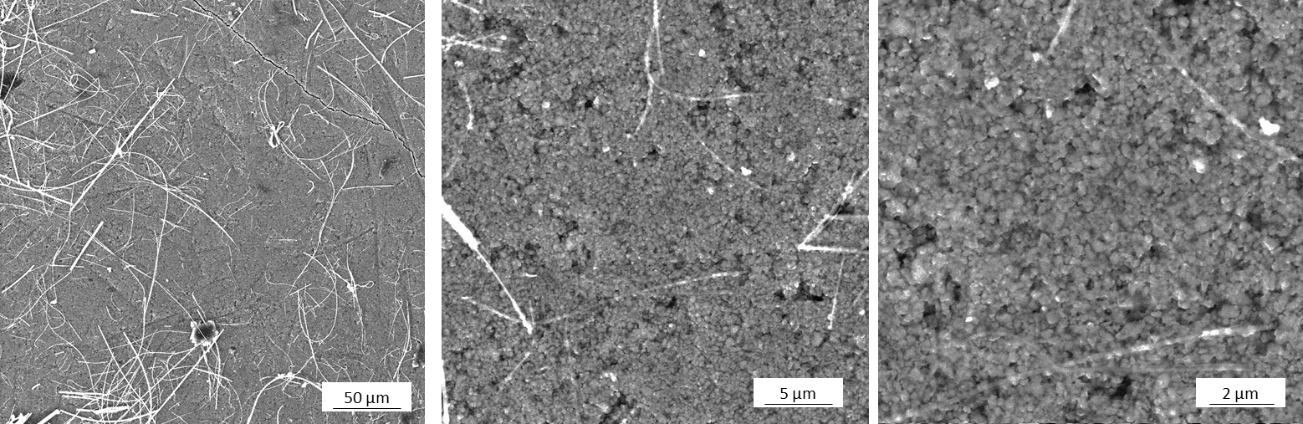

2.
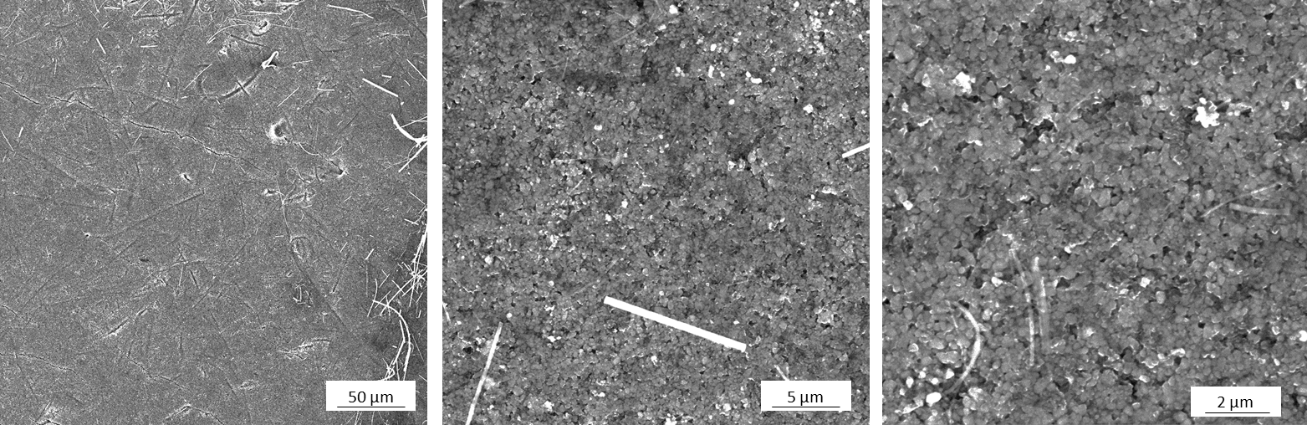


**Figure S18.** SEM images collected after cycling: a) 85 wt.% LFMP46-15 wt.% PEDOT:PSSTFSI and b) 85 wt.% LFMP46 - 15 wt.% PVDF:CB (1:1)

In Figure S18, fibrous components were observed, originating from the Whatman separator. This is due to the incomplete separation between the positive electrode and the separator.

- 1. **Electrochemical results**
     1. *Rate capability tests with LiFe_0.4_Mn_0.6_PO_4_ (LFMP46) as positive electrode material*

1. LFMP46 with PEDOT:PSSTFSI as mixed ionic and electronic conductor


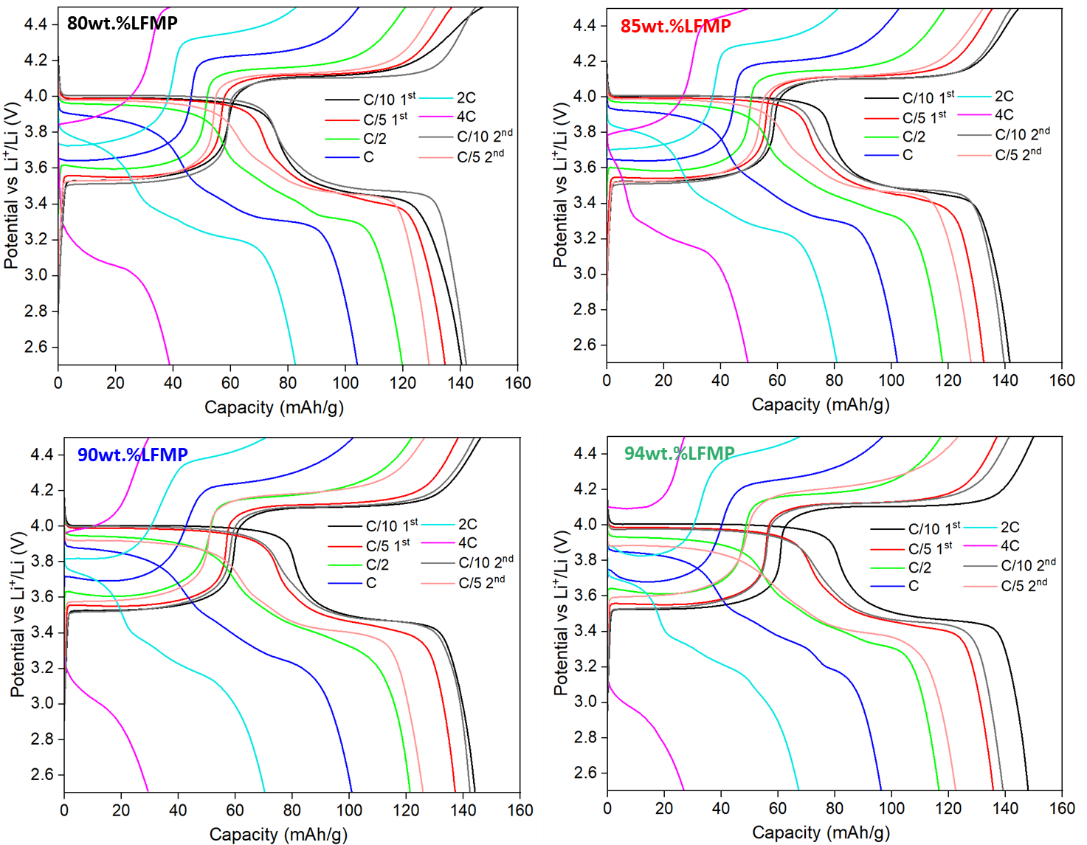


1. LFMP46 with PVDF as binder and carbon black as conductive additive


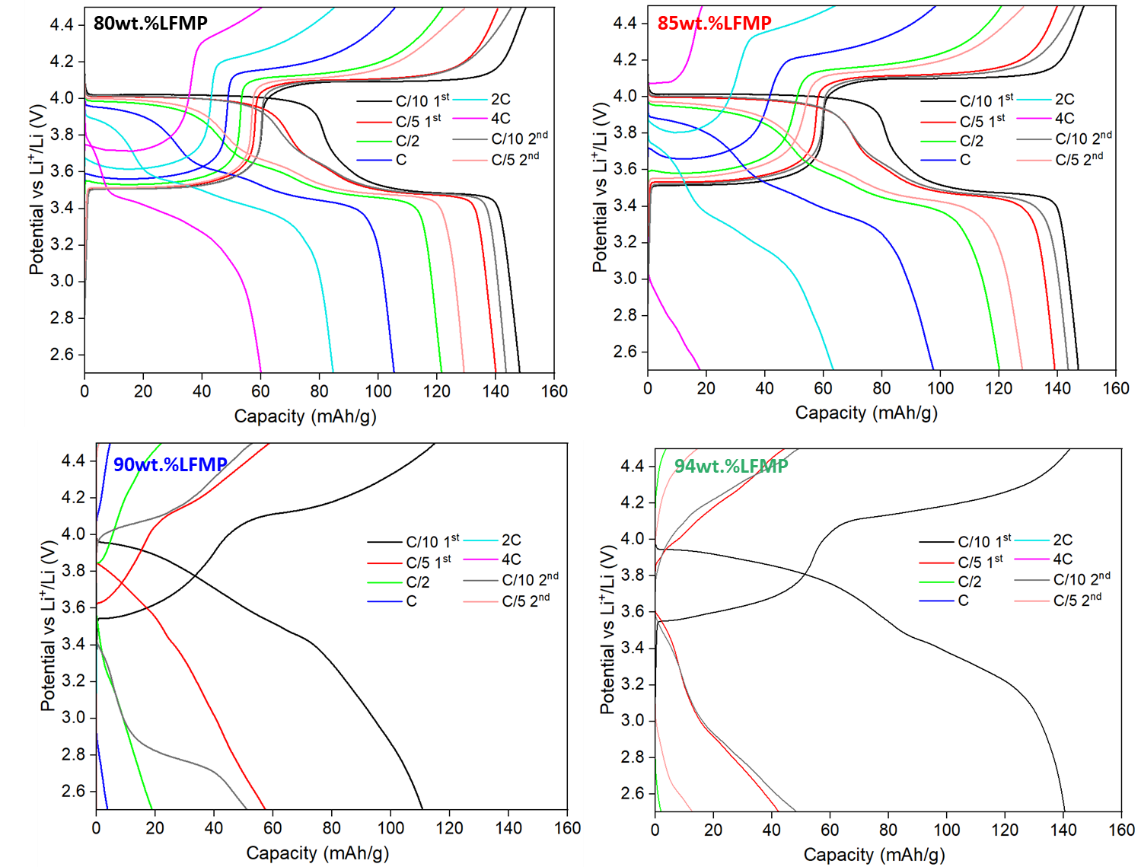


**Figure S19.** GCPL results for a) LFMP46-PEDOT:PSSTFSI and b) LFMP46-PVDF:CB at various C-rates

The results of GCPL experiments for composite electrodes made of LiFe_0.4_Mn_0.6_PO_4_ (LFMP46) and PEDOT:PSSTFSI reveal similar electrochemical performance whatever the loading in active material, and a huge improvement is observed compared to the reference electrode formulation (with PVDF and carbon black), especially for compositions rich in active material. The slight increase of capacity compares to the reference indicates better overall electrochemical performance due to superior electronic conductivity of PEDOT:PSSTFSI and better swellability, helps more efficient electron transfer process and extraction-insertion of lithium ions. The performance of all batteries is compared as follows:

**Figure S20**. GCPL results for LFMP46 samples upon cycling at various C-rates and using different electrode formulations

- - 1. *Apparent Lithium diffusion coefficient determination*

The apparent lithium diffusion coefficient was determined by the Randles–Sevcik equation as follows^[12,13]^:

$$\frac{i_{p}}{m}=0.4463F\left( \frac{F}{RT} \right)^{1/2}C_{Li}^{*}v^{1/2}A_{e}D_{app}^{1/2}$$

Where *m* represents the electrode's active material mass, $C_{Li}^{*}$ the initial concentration of lithium in LiFe_0.4_Mn_0.6_PO_4_, $A_{e}$ the electrode area per mass unit, and $D_{app}$ the apparent diffusion constant of lithium. $C_{Li}^{*}$ is considered as the total quantity of lithium within a particle before delithiation of LiFe_0.4_Mn_0.6_PO_4_, whose bulk density is of 3.4 g/cm^3^ and molar mass of 157.25 g/mol, leading to a lithium concentration of 0.0216 mol/cm^3^.

The results of the linear fits are given below:

**Table S5.** Results from Cyclic Voltammetry linear fits

| **Item** | **Line** | **Linear fit equation** | **R^2^** |
| --- | --- | --- | --- |
| 85 wt.% LFMP46-15 wt.% PEDOT:PSSTFSI | Cathodic | y = 20.314x – 0.0535 | 0.9992 |
|  | Anodic | y = 16.55x – 0.0952 | 0.9805 |
| 85 wt.% LFMP46-15 wt.% PVDF:carbon black (1:1) | Cathodic | y = 9.2705x + 0.0021 | 0.9977 |
|  | Anodic | y = 8.3691x – 0.0162 | 0.9933 |

- - 1. *Electrode porosity results*

The result of the electrode component density measurements using Helium pycnometer is shown as follow:

**Table S6.** Density of electrode components obtained using Helium pycnometer

| Components | Pycnometric density (g/mL) |
| --- | --- |
| LiFe_0.4_Mn_0.6_PO_4_ | 3.50 |
| PVDF | 1.78 |
| Carbon Black | 1.85 |
| Dry PEDOT:PSSTFSI | 2.47 |

While the result of electrode porosity is shown as follow:

**Table S7.** Electrode porosity measurement

| Sample | Mass of electrode (mg) | Electrode thickness (µm) | Electrode volume (µL) | Experimental electrode density (g/mL) | Theoretical electrode density (g/mL) | Electrode porosity (%) | Average porosity (%) |
| --- | --- | --- | --- | --- | --- | --- | --- |
| LFMP-PEDOT:PSSTFSI | 9.0 | 16.8 | 3.38 | 2.7 | 3.3 | 19 | 19 |
|  | 9.3 | 17.0 | 3.42 | 2.7 |  | 17 |  |
|  | 7.6 | 14.7 | 2.95 | 2.6 |  | 22 |  |
| LFMP-REFERENCE | 6.3 | 12.0 | 2.41 | 2.6 | 3.1 | 15 | 16 |
|  | 9.0 | 17.0 | 3.42 | 2.6 |  | 14 |  |
|  | 9.2 | 18.1 | 3.64 | 2.5 |  | 18 |  |

Both LFMP-PEDOT:PSSTFSI and LFMP reference have similar electrode thickness and porosity. Therefore, as it has similar microstructure properties including electrode thickness, porosity, particle size and distribution (as revealed by SEM images) etc., we could conclude that the higher lithium diffusion in LFMP46-PEDOT:PSSTFSI electrode essentially originates from the higher ionic conductivity of PEDOT:PSSTFSI provided by the TFSI entities.

- - 1. *Polarization evolution upon cycling (dQ/dE)*

The evolution of dQ/dE over the first 50^th^ cycles obtained at a C/5 rate is shown as follow:

**Figure S21.** The evolution of dQ/dE over the first 50^th^ cycles obtained at a C/5 rate.

The two cathodic peaks correspond to the two voltage domains associated to the redox activity of Fe^3+^/Fe^2+^ at around 3.5 V vs. Li^+^/Li and of Mn^3+^/Mn^2+^ at around 4.1 V vs. Li^+^/Li. The polarization corresponds to the difference in voltage between the cathodic and anodic peaks.

Evolution of the polarization upon cycling over 150 cycles performed at C/5 for 85 wt.% LFMP46 loaded electrode:

| 1. 85 wt.% LFMP46-15 wt.% PEDOT:PSSTFSI    | 1. 85 wt.% LFMP46-15 wt.% PVDF:carbon black (1:1) |
| --- | --- |

**Figure S22**. Evolution of dQ/dE over 150 cycles performed at C/5 rate for a) 85 wt.% LFMP46-15 wt.% PEDOT:PSSTFSI and for b) 85 wt.% LFMP46-15wt.% PVDF:carbon black (1:1)

For clearer observation and comparison between LFMP46-PEDOT:PSSTFSI and LFMP46-PVDF:CB reference, the dQ/dE derivatives are compared for the 1^st^, 5^th^, 25^th^, 50^th^, 100^th^, and 150^th^ cycles.

|  |  |
| --- | --- |
|  |  |
|  |  |
|  |  |

**Figure S23**. The evolution of dQ/dE for LFMP46-PEDOT:PSSTFSI electrode (sample) and LFMP46-PVDF:CB (1:1) reference electrode (reference) over 150 cycles at the C/5 rate

- 1. **Electrochemical performance of LFMP46 reference electrode without carbon black addition**

To compare the binder performance of PEDOT:PSSTFSI with those of PVDF, a series of electrochemical tests was also carried out for LFMP46 with PVDF only for the 85 wt.% LFMP46, without any addition of carbon black.

- - 1. *Long cycling test*

a) b)

**Figure S24.** a) GCPL results and b) evolution of discharge capacity over 150 cycles performed at C/5 rate

- - 1. *Impedance test*

**
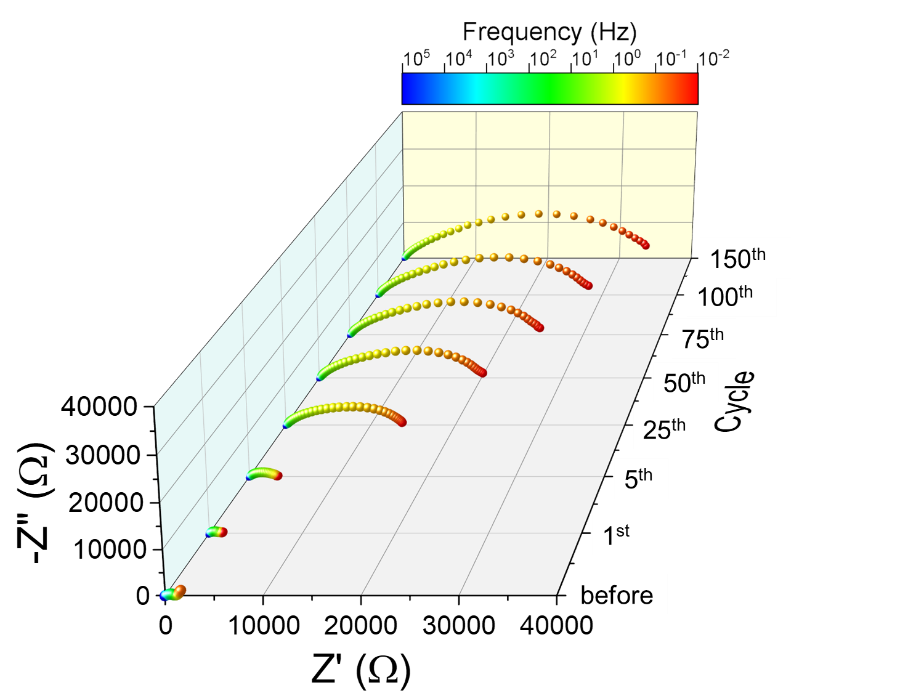
**

**Figure S25**. Impedance spectra of LFMP46 reference electrode using PVDF as binder but without addition of carbon black as conductive additive

References

[1] A. Hofmann, *Thesis, Université de Bordeaux,* **2016**.

[2] H. Topsoe, *Semiconductor Division,* **1966**.

[3] A. Chelly, S. Glass, J. Belhassen, A. Karsenty, *Results Phys.* **2023**, *48*, 106445.

[4] M. B. McDonald, P. T. Hammond, *ACS Appl. Mater. Interfaces* **2018**, *10*, 15681.

[5] W. J. Albery, Z. Chen, B. R. Horrocks, A. R. Mount, P. J. Wilson, D. Bloor, A. T. Monkman, C. M. Elliott, **1989**, 247.

[6] P. G. Pickup, *J. CHEM. SOC. FARADAY TRANS.* **1990**, *86*, 3631.

[7] Q. D. Nguyen, E. S. Oh, K. H. Chung, *Polym. Test.* **2019**, *76*, 245.

[8] M. Kocun, A. Labuda, W. Meinhold, I. Revenko, R. Proksch, *ACS Nano* **2017**, *11*, 10097.

[9] G. Pace, A. Zele, P. Nguyen, J. Cl, R. A. Segalman, *Chem. Mater. ,***2023**.

[10] R. Meziane, J. P. Bonnet, M. Courty, K. Djellab, M. Armand, in *Electrochim. Acta*, Elsevier Ltd, **2011**, pp. 14–19.

[11] H. Yousefian, S. A. Hashemi, A. Babaei-Ghazvini, B. Acharya, A. Ghaffarkhah, M. Arjmand, *Mater. Adv.* **2024**, *5*, 4699.

[12] D. Y. W. Yu, C. Fietzek, W. Weydanz, K. Donoue, T. Inoue, H. Kurokawa, S. Fujitani, *J. Electrochem. Soc.* **2007**, *154*, A253.

[13] G. T. Pace, M. L. Le, R. J. Clément, R. A. Segalman, *ACS Energy Lett.* **2023**, *8*, 2781.
